# Human Neural Progenitors Expressing GDNF Enhance Retinal Protection in a Rodent Model of Retinal Degeneration

**DOI:** 10.1093/stcltm/szad054

**Published:** 2023-10-03

**Authors:** Saba Shahin, Patrick Tan, Jason Chetsawang, Bin Lu, Soshana Svendsen, Stephany Ramirez, Trevor Conniff, Jorge S Alfaro, Michael Fernandez, Aaron Fulton, Alexander H Laperle, Clive N Svendsen, Shaomei Wang

**Affiliations:** Board of Governors Regenerative Medicine Institute, Department of Biomedical Sciences, Cedars-Sinai Medical Center, Los Angeles, CA, USA; Board of Governors Regenerative Medicine Institute, Department of Biomedical Sciences, Cedars-Sinai Medical Center, Los Angeles, CA, USA; Board of Governors Regenerative Medicine Institute, Department of Biomedical Sciences, Cedars-Sinai Medical Center, Los Angeles, CA, USA; Board of Governors Regenerative Medicine Institute, Department of Biomedical Sciences, Cedars-Sinai Medical Center, Los Angeles, CA, USA; Board of Governors Regenerative Medicine Institute, Department of Biomedical Sciences, Cedars-Sinai Medical Center, Los Angeles, CA, USA; Board of Governors Regenerative Medicine Institute, Department of Biomedical Sciences, Cedars-Sinai Medical Center, Los Angeles, CA, USA; Board of Governors Regenerative Medicine Institute, Department of Biomedical Sciences, Cedars-Sinai Medical Center, Los Angeles, CA, USA; Board of Governors Regenerative Medicine Institute, Department of Biomedical Sciences, Cedars-Sinai Medical Center, Los Angeles, CA, USA; Board of Governors Regenerative Medicine Institute, Department of Biomedical Sciences, Cedars-Sinai Medical Center, Los Angeles, CA, USA; Board of Governors Regenerative Medicine Institute, Department of Biomedical Sciences, Cedars-Sinai Medical Center, Los Angeles, CA, USA; Board of Governors Regenerative Medicine Institute, Department of Biomedical Sciences, Cedars-Sinai Medical Center, Los Angeles, CA, USA; Board of Governors Regenerative Medicine Institute, Department of Biomedical Sciences, Cedars-Sinai Medical Center, Los Angeles, CA, USA; David Geffen School of Medicine, Department of Medicine, University of California Los Angeles, Los Angeles, CA, USA; Board of Governors Regenerative Medicine Institute, Department of Biomedical Sciences, Cedars-Sinai Medical Center, Los Angeles, CA, USA; David Geffen School of Medicine, Department of Medicine, University of California Los Angeles, Los Angeles, CA, USA

**Keywords:** neural progenitors, retinal degeneration, visual function, trophic factors, oxidative stress

## Abstract

Stem cell therapy for retinal degenerative diseases has been extensively tested in preclinical and clinical studies. However, preclinical studies performed in animal models at the early stage of disease do not optimally translate to patients that present to the clinic at a later stage of disease. As the retina degenerates, inflammation and oxidative stress increase and trophic factor support declines. Testing stem cell therapies in animal models at a clinically relevant stage is critical for translation to the clinic. Human neural progenitor cells (hNPC) and hNPC engineered to stably express GDNF (hNPC^GDNF^) were subretinally injected into the Royal College of Surgeon (RCS) rats, a well-established model for retinal degeneration, at early and later stages of the disease. hNPC^GDNF^ treatment at the early stage of retinal degeneration provided enhanced visual function compared to hNPC alone. Treatment with both cell types resulted in preserved retinal morphology compared to controls. hNPC^GDNF^ treatment led to significantly broader photoreceptor protection than hNPC treatment at both early and later times of intervention. The phagocytic role of hNPC appears to support RPE cell functions and the secreted GDNF offers neuroprotection and enables the extended survival of photoreceptor cells in transplanted animal eyes. Donor cells in the RCS rat retina survived with only limited proliferation, and hNPC^GDNF^ produced GDNF in vivo. Cell treatment led to significant changes in various pathways related to cell survival, antioxidative stress, phagocytosis, and autophagy. A combined stem cell and trophic factor therapy holds great promise for treating retinal degenerative diseases including retinitis pigmentosa and age-related macular degeneration.

## Teaser

This study examined vision protection by human neural progenitor cells (hNPC) and hNPC engineered to express GDNF (hNPC^GDNF^) in a rat model of retinal degeneration at a disease stage relevant to clinical translation. Compared to hNPC alone, hNPC^GDNF^provided enhanced vision protection, as well as significantly broader photoreceptor protection when intervention was at early and later stages of retinal degeneration. Several potential mechanisms include activating survival pathways, reducing oxidative stress, and mediating autophagy and phagocytosis. These findings, along with the safety profile from clinical studies, indicate that hNPC^GDNF^could be used to treat various retinal degenerative diseases.

## Graphical Abstract

**Graphical Abstract:**
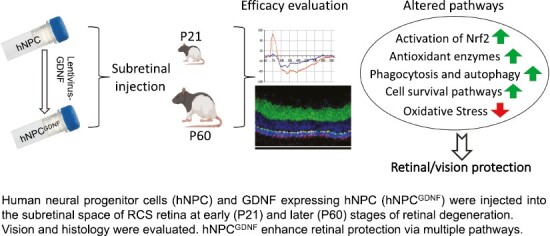

## Introduction

Vision loss has a tremendous and life-changing impact on people worldwide.^1,2^ Retinitis pigmentosa (RP) is the most common inherited retinal disease of the various retinal defects that lead to progressive degeneration of light-sensitive rod and cone photoreceptors, ultimately with irreversible vision loss.^3-5^ According to RetNet (https://sph.uth.edu/retnet/), at least 71 genes have been associated with syndromic and non-syndromic RP. Currently, it is estimated that RP affects around 1 in 4000 people in the world. Degeneration in RP, which leads to tunnel vision and finally blindness,^6^ can be divided into 4 progressive stages: peripheral rod photoreceptors start to degenerate toward the center of the retina (stage I); loss of rods causes degeneration of cone outer segments from the periphery toward the center of the retina (stage II); and cone photoreceptors lose outer segments (stage III). In the final stage of degeneration (stage IV), cone cell bodies may completely degenerate.^7^ Most patients present to the clinic at stage II, at which point slowing down retinal degeneration should be the key focus for research and treatment.

Treatment options for RP remain limited. Gene therapy for a single mutation has shown effectiveness in treating recessive RP.^5,8^ In 2017, the US FDA approved Luxturna, a novel RPE65 gene replacement therapy to treat patients with autosomal recessive RP caused by RPE65 mutation.^9^ But, to date, long-term efficacy from clinical reports has been controversial.^5,10-12^ In addition, the RPE65 mutation only accounts for a small percentage of RP cases. Due to this fact, along with the genetic diversity of RP, mutation-independent treatments are needed.

Advances in stem cell biology have offered promise for treating retinal degeneration as a preservation strategy, which would be an option irrespective of the mutation. Currently, there are clinical trials using stem cell therapy for retinal degeneration.^13-15^ However, positive results from preclinical studies were not reflected in clinical outcomes. This may largely be because most preclinical studies were performed at a very early stage of degeneration when the retinal environment is still relatively healthy. During progressive retinal degeneration there is increased inflammation and oxidative stress as well as trophic factor deprivation. This hostile retinal environment may require more than only stem cell delivery.

Various neurotrophic and other growth factors have provided promising rescue of photoreceptors and vision protection in animal models and clinical trials for several retinal neurodegenerative diseases.^16-20^ In particular, glial cell line-derived neurotrophic factor (GDNF) can slow down photoreceptor degeneration in numerous animal models of retinal degeneration including RP.^21-23^ GDNF can provide direct protection of photoreceptors, which express specific GDNF receptors^24^ and can indirectly protect photoreceptors via activation of retinal Müller glia, which then increase production of basic fibroblast growth factor, brain-derived neurotrophic factors and GDNF.^25^ It has been reported that multiple growth factors together offer enhanced retinal protection compared to a single factor.^18^

The Royal College of Surgeon (RCS) rat is a well-established model of RP in which a mutation in the *MERTK* gene leads to the inability of RPE to phagocytose shed photoreceptor outer segments (POS). Accumulation of POS debris between the outer nuclear layer (ONL) and RPE prevents the supply of essential nutrients from the choroid vessels to photoreceptors, leading to their degeneration and eventual death.^26-28^As with human RP, progressive retinal degeneration in RCS rats can be classified into 4 stages,^29–32^ although retinal degeneration rate is much faster in the animal model. At stage I, postnatal day (P) 21-23, rats have a vision similar to wild-type, but rod function is compromised. At stage II (P40-43), rod photoreceptors and function have degenerated substantially, cone photoreceptors are still preserved with inner and outer segments, and cone function is normal. At stage III (P60-63), both rod and cone morphology and function have been compromised, but cone function is still less affected, and intervention at this stage is to protect cone function. Stages II-III of degeneration in the animal model are similar to the clinical stage II of RP in human patients and hence are most relevant for testing a cell therapy that would ideally delay photoreceptor degeneration and preserve cone function. Stage IV (>P100) has severely compromised rod and cone morphology and function, and with a single layer of cone remaining, preservation therapy would likely be ineffective.

Based on our previous studies and further investigational new drug (IND)-enabling studies, clinical-grade hNPC (termed CNS10-NPC) are being delivered to the subretinal space of patients with RP in a current phase I/2a clinical trial to assess safety (NCT04284293). We have further shown that hNPC-secreting GDNF (hNPC^GDNF^) can augment the preservation of visual function; however, an intervention was at an early stage of the disease.^33^ To be clinically relevant, it is necessary to assess the ability of hNPC and hNPC^GDNF^ to preserve vision at a later stage of degeneration.

In this study, RCS rats received subretinal injections of hNPC and hNPC^GDNF^ at early and later stages of retinal degeneration. We demonstrated that, compared to hNPC, hNPC^GDNF^ offers significantly more preservation of photoreceptors and vision, indicating that a combined stem cell and gene therapy approach may provide a better outcome in treating RP. The protective effect involves several mechanisms, which include activating survival pathways, reducing oxidative stress, and mediating autophagy and phagocytosis. This is the first demonstration that a neural progenitor cell and growth factor-based treatment is effective in the clinically relevant stage of disease. The next step is additional IND-enabling studies to bring hNPC^GDNF^ to the clinic for treating retinal degenerative diseases.

## Methods and Materials

### Animals

**Figure 1.**
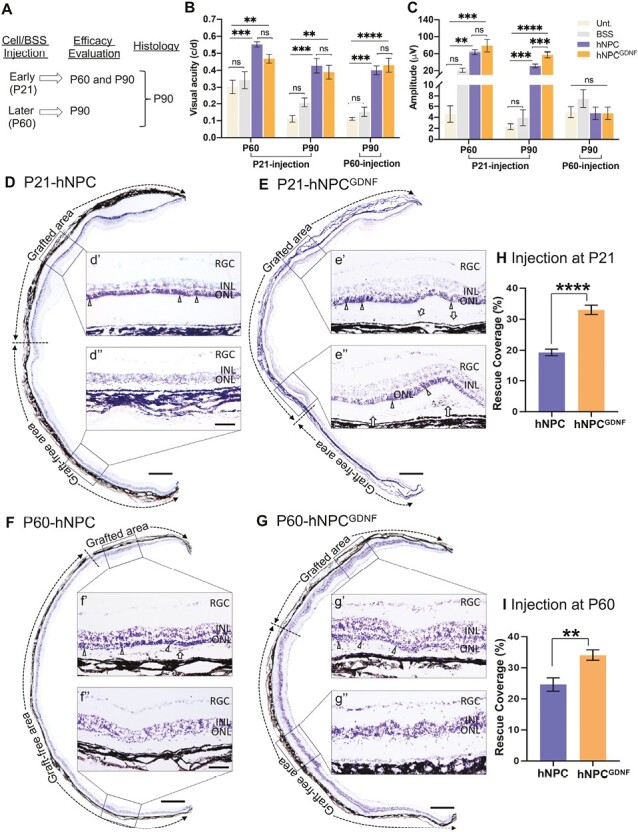
hNPC and hNPC^GDNF^ preserve vision. **(A)** Timeline shows injection of cells or BSS at early (P21) and later (P60) time points, followed by testing efficacy at P60 and P90 and retinal histology at P90. **(B)** Optokinetic response shows significantly increased visual acuity after hNPC and hNPC^GDNF^ injection at P21 or P60 compared to untreated and BSS controls. **(C)** ERG shows significantly higher b-wave amplitude with both hNPC and hNPC^GDNF^ injections at P21 compared to untreated and BSS controls. Note, hNPC^GDNF^-treated eyes scored a significantly higher b-wave amplitude than hNPC-treated eyes at the P90 time point. No significant change in the b-wave amplitude was observed among groups after P60-injection. Data are represented as mean ± SEM (*n* = 8-15). One-way ANOVA with Tukey’s test was used for multiple comparisons. **(D-G)** Montage of cresyl violet-stained retina shows that P21 cell injection led to 6-8 layers of photoreceptor nuclei with hNPC **(D)** and hNPC^GDNF^**(E)** in the graft-protected area compared to the area away from the graft (graft-free area) showing a single layer of photoreceptor nuclei. For P60-injection, the treated areas showed 3-5 layers of photoreceptor nuclei with hNPC **(F)** and hNPC^GDNF^**(G)**. Graft-protected and graft-free areas are demarcated by bi-headed dotted arrows. dʹ-g″ are high-power images of the corresponding outlines; arrows point to potential donor cells in eʹ, e″, and fʹ; triangles point to ONL. **(H&I)** Quantification of percent rescue coverage of photoreceptor length shows photoreceptor preservation in hNPC- and hNPC^GDNF^-treated retina. Note, significantly increased percent rescued coverage of photoreceptor length in retinas treated at P21 and P60 time points with hNPC^GDNF^ compared to hNPC. Scale bar = 800 μm for the retinal montage images and 100 μm for high power images. Unt, untreated; BSS, balanced salt solution; INL, inner nuclear layer; ONL, outer nuclear layer; RGC, retinal ganglionic cell. Data are represented as mean ± SEM (*n* = 5-6). Student’s 2-tailed *t*-test. **P ≤ .01, ***P ≤ .001, ****P ≤ .0001, ns: nonsignificant.

Experimental groups included group 1 (*n* = 29) that received a subretinal injection of either hNPC^GDNF^ (*n* = 15) or hNPC (*n* = 14) in one eye at P21-23 (stage I of RP) and included group 2 (*n* = 21) that received subretinal injection of hNPC^GDNF^ (*n* = 8) and hNPC (*n* = 13) at P60 (stage II/III of RP; Fig. 1A). Contralateral eyes served as controls that either received balanced salt solution (BSS) or were untreated. We have previously shown that BSS and untreated controls are functionally and morphologically comparable.^34,35^ Cyclosporine A was used in this xenograft animal model to reduce the immune response following the injection of human-derived cells. Animals were administered cyclosporine A in the drinking water (210 mg/L) from 1 day prior to transplantation until sacrifice. All rats were sacrificed at P90 following the Cedars-Sinai Medical Center’s Institutional Animal Care and Use Committee (IACUC 7611) and the ARVO Statement for the Use of Animals in Ophthalmic and Vision Research.

### Cell Source, Preparation, and Transplantation

The generation and expansion of human neural progenitors have been previously described.^36,37^ hNPC (termed G010 cell line) were derived from an 8-week-old fetal cortex and expanded in culture with recombinant human epidermal growth factor (rhEGF, 100 ng/mL) and leukemia inhibitory factor (rhLIF, 100 ng/mL) as free-floating neurospheres, which were passaged using a chopping method. hNPC^GDNF^ was created using lentivirus transduction according to our published protocol.^38^ Research-grade hNPC and hNPC^GDNF^ were used in this study, under the Stem Cell Research Oversight Committee (Pro00025772) at Cedars-Sinai Medical Center.

Cells were prepared according to our published protocol.^35^ Briefly, after removal from liquid nitrogen, hNPC and hNPC^GDNF^ vials were thawed quickly in a water bath at 37 °C and resuspended in prewarmed Stemline media (Sigma, St. Louis, Missouri) supplemented with rhEGF (100 ng/mL; Millipore, Billerica, MA) and rhLIF (100 ng/mL; Millipore). The cell suspension was centrifuged at 200*g* for 5min, resuspended in BSS (Alcon) followed by centrifugation and resuspension at 30 000 cells/µL in BSS. Cells were kept on ice until transplantation.

Cell injection was performed with our previous protocol.^35^ Briefly, 2 µL suspension of hNPC or hNPC^GDNF^ (6 × 10^4^ cells/eye) was delivered into the subretinal space through a small scleral incision with a fine glass pipette (internal diameter, 75-150 μm) attached by tubing to a 25-μL syringe (Hamilton). The cornea was punctured to reduce intraocular pressure and to limit the efflux of cells. Contralateral eyes serving as controls received 2 µL of BSS or were untreated. Immediately after injection, the fundus was examined for retinal damage or signs of vascular distress. Any animals showing such problems were excluded from further study.

### Efficacy Evaluation

#### Optokinetic Response (OKR)

OKR offers non-invasive screening to detect spatial visual acuity measured in cycle/degree (c/d). Visual acuity was tested at P60 and P90 by OKR based on our published protocol.^33,35^ The OKR was observed and recorded by 2 blinded investigators.

#### Electroretinography (ERG)

ERG measures the average of whole retinal activities to light simulation. ERG was conducted at P60 and P90 based on our previous protocol.^35,39^ The eye was stimulated with full-field light flashes by a computer-controlled system using the Espion system (Diagnosys LLC). A total of 20-30 sweeps for each animal were recorded, and the average responses were used as the response amplitude.

### Tissue sampling

All eyes were collected and processed at P90. In Group 1, RCS rats (*n* = 29) received hNPC (*n* = 14) or hNPC^GDNF^ (*n* = 15) in one eye at postnatal day 21-23. For histology and immunohistochemistry, 5 eyes were processed for the hNPC group and 6 eyes were processed for the hNPC^GDNF^ group. From each cell treatment group, 4 eyes were used for RNA isolation and 4 eyes were used for protein extraction. One remaining eye from each treatment group was processed for protein extraction and stored if needed. In Group 2, RCS rats (*n* = 21) received hNPC (*n* = 13) or hNPC^GDNF^ (*n* = 8) in one eye at postnatal day 60. From this group, all eyes were processed for histology and immunohistochemistry.

For RNA isolation and western blotting, half of the retina containing the injection site (dorsal-temporal part) was dissected. For BSS and untreated retinas, the dorsal-temporal part of the retina at the same orientation as the cell-treated eye was dissected. Retinas were stored at −80 °C for western blotting and the rest was immediately processed for RNA isolation and cDNA synthesis.

### Histology

Eyes were enucleated, fixed in 4% paraformaldehyde, and embedded in OCT for cryostat sections. Retinal cryostat sections were placed on a series of 5 slides, with 4 sections per slide. The first slide of each series was used for cresyl violet staining to visualize retinal lamination and donor cell location and for photoreceptor quantification. The remaining slides were used for antibody staining following previously described protocols.^39^ Images were taken with Leica microscopy.

#### Quantification of Photoreceptor Preservation

Retinal montage images were taken on cresyl violet stained retinal sections from both hNPC- and hNPC^GDNF^-treated rats at early and later time points and processed to quantify rescue coverage (%) to determine photoreceptor preservation. Briefly, the length of ONL with more than 2 layers of photoreceptors and the whole retinal length on retinal sections were measured in 6-8 sections/eye, 5-6 eyes/cell treatment/time points using “freehand lines” tool of Java-based image processing software (ImageJ). The percentage of ONL preservation against the whole retinal length was calculated by dividing the protected length of ONL (with more than 2 layers of photoreceptors) by the total retinal length to quantify rescue coverage (%). Since both BSS-treated and untreated eyes at this time have a single layer of photoreceptors, only cell-treated eyes were used for quantification analysis.

#### ABC Staining

Slides were washed in phosphate-buffered saline (PBS) before permeabilization and blocked in a solution containing 0.2% Triton-X 100 and 2% horse serum in PBS for 1 h at room temperature. Sections were incubated overnight in GDNF antibody (1:1000, Thermo Fisher Scientific) in a blocking solution. Slides were then washed with PBS and incubated with the secondary antibody for an hour. A vector avidin/biotin complex (ABC; Vector Laboratories) solution was prepared per manufacturer instruction and applied for 1 h before washing with PBS. Slides were then incubated with nickel-intensified diaminobenzidine for 3-5 min, and washed in PBS. Finally, slides were mounted using a DPX mounting solution.

#### Immunofluorescent Staining

Retinal sections were stained with the primary antibodies (Supplementary Table S1) with our published protocols.^35,39^ Anti-mouse or rabbit secondary antibodies conjugated to Alexa Fluor-488 or Alexa Fluor-568 (Life Technologies) were used and counterstained with 49,69-diamidino-2-phenylindole (DAPI) before mounting slides using Fluorescent Mount media (Sigma-Aldrich).

### RNA Extraction and qPCR

Total RNA was extracted from retinas using a QIAGEN RNeasy Mini kit (Qiagen Hilden, Germany). cDNA was synthesized in a 20 μL reaction using 500 ng total RNA and ProtoScript II First Strand cDNA Synthesis Kit (New England Biolabs Inc.). Primers were synthesized by Integrated DNA Technologies (Supplementary Table S2). Quantitative real-time PCR was performed with PowerUp SYBR Green Master Mix (Applied Biosystems). The transcript levels of target genes were assessed using Bio-Rad CFX384 Real-Time PCR Detection System (Bio-Rad, 1 855 485). Cycling parameters were: 95 °C for 2 minutes, 95 °C for 15 s, 60 °C for 15 s, and 72 °C for 30 s, with 40 cycles of amplification. The mRNA levels were normalized to the corresponding *GADPH* values. Differential expression was determined using the deltaCt method.

### Western Blot

Retinas were lysed with RIPA buffer and protein was quantified using a BCA protein assay (Pierce). Protein of 50 μg from each group was resolved on 4%-20% precast polyacrylamide gels (catalog 4561094, Bio-Rad) and transferred to nitrocellulose blots. Blots were incubated in 2.5% BSA blocking solution for 1 h followed by primary antibodies (Supplementary Table S1) incubation overnight. Signals were detected using fluorescent secondary antibodies and the Odyssey imager from LI-COR. Densitometric analysis was performed using Image Studio Acquisition Software and relative expression was determined by normalizing with GAPDH.

### Data Analysis

Statistical analyses were performed by using GraphPad Prism software (version 9.3). Data are presented as mean ± SEM. Results were analyzed by unpaired 2-tailed Student’s *t*-test for 2-group comparisons and 1-way ANOVA coupled with a Tukey’s multiple comparisons test for more than 2-group. *P* value < .05 was considered significant.

## Results

### hNPC and hNPC^GDNF^ Treatment Preserve Visual Function

Wild-type rats have a visual acuity around 0.55c/d^40^ and the RCS rat’s visual acuity deteriorates with time. Visual acuity was measured at P60 and P90 for animals with injection at P21 and P90 for later time injection (Fig. 1A). Visual acuity was significantly higher with treatment at the early (P21) and later (P60) stages with hNPC and hNPC^GDNF^ compared to BSS-treated and untreated controls (Fig. 1B). There is no significant difference between hNPC and hNPC^GDNF^-treated eyes at both P60 and P90 time points. As expected, visual acuity deteriorates from P60 to P90 in control-treated eyes. However, of note, visual acuity remained stable over time regardless of whether cell injection was at P21or P60 (*P* > .05). Following cell treatment at P21, ERG b-wave was significantly higher compared to controls at both P60 and P90 time points (Fig. 1C; Supplementary Fig. S1). Notably, treatment with hNPC^GDNF^ provided significantly higher ERG b-wave compared with hNPC at P90. There was no difference among groups at the later time of intervention.

### hNPC and hNPC^GDNF^ Treatment Preserves Retinal Cells

Following vision tests at P90, animals were euthanized and processed for histology (Fig. 1A). Cresyl violet-stained retinal sections from P90 showed photoreceptor preservation with cells at both P21 and P60 injection, with perseveration regions associated with donor cell distribution (Fig 1D–G). In contrast, regions distal to the donor cells had only a single layer of photoreceptors remaining, which was also the case for controls. hNPC^GDNF^ provided significantly more rescue coverage compared to hNPC at both early (Fig. 1H, 33.92% vs. 22.68%) and later (Fig. 1I, 33.02% vs. 19.26%) treatment time points. Notably, the percentage of photoreceptor preservation in hNPC^GDNF^-treated eyes was approximately 33% at both early and later time treatments.

**Figure 2.**
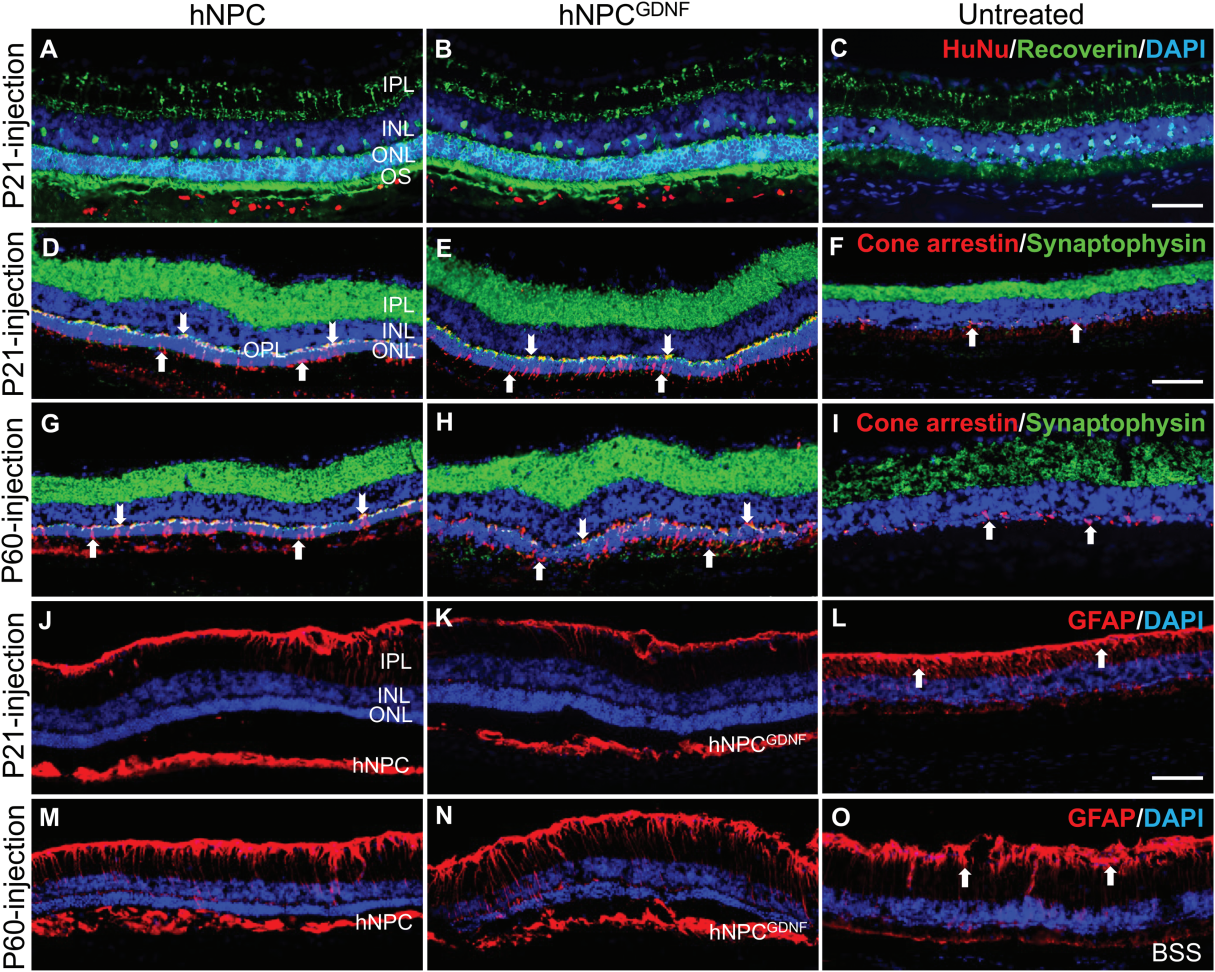
hNPC and hNPC^GDNF^ preserve photoreceptors and retinal synapses as well as suppress Müller cell gliosis. (**A-C**) Double immunostaining of recoverin and human-specific nuclear marker revealed organized photoreceptor outer segments (OS) and ONL preservation in both hNPC- and hNPC^GDNF^-treated retina. In contrast, untreated retina showed a single layer of degenerating photoreceptors. **(D-I)** Double immunostaining of cone arrestin and synaptophysin showed cones with organized cell bodies (arrows), inner and outer segments, and pedicles (chevron) along with preserved synapses in IPL and OPL in retinal regions treated with cells at P21 **(D&E)** or P60 **(G&H)**. In comparison, the untreated retina **(F)** and area away from graft **(I)** showed a discontinuous layer (arrows) of degenerated cones without segments, and pedicles as well as reduced synaptic density in IPL and OPL for both P21- and P60-injection. **(J-O)** Immunostaining of GFAP showed reduced Müller cell activation/gliosis in retinas treated at P21 with cells **(J&K)** compared to untreated **(L**, arrows). More active Müller glia were observed after P60 cell injection **(M&N)** compared to retinas treated at P21, with similar levels to the BSS control retina **(O)**. Both hNPC and hNPC^GDNF^ showed GFAP staining in the subretinal space of the retina for both early and later intervention (triangles). DAPI counterstain in blue. Scale bar = 100 μm. OS, outer segment; ONL, outer nuclear layer; INL, inner nuclear layer; IPL, inner plexiform layer; OPL, outer plexiform layer.

To examine whether hNPC and hNPC^GDNF^ can preserve photoreceptors (rods and cones) and retinal connections, double immunostaining was performed on retinal sections for recoverin (green) and the human-specific nuclear marker (HuNu, red; Fig. 2A–C; Supplementary Fig. S2A–S2C), along with cone arrestin (red) and synaptophysin (green; Fig. 2D–2I). Recoverin immunostaining showed morphologically preserved photoreceptors with improved POS length (Fig. 2A–2C; Supplementary Fig. S2A–S2C) in the grafted area of both the hNPC- and hNPC^GDNF^-treated retina compared to the region distal from the graft and the untreated retina, which showed a single layer of degenerating photoreceptors with diminished POS. Further, cone preservation with organized inner and outer segments and pedicles was observed in the area associated with donor cell distribution. In contrast, the area distal to the cell injection site and the untreated retina had cones that were distributed as a discontinuous layer, with no segments and pedicles. Correlated with photoreceptor preservation, both inner and outer plexiform layers (OPL) revealed by synaptophysin, were preserved in cell-treated retinas; while in controls, the OPL (connection between ONL and INL) was hardly visible, indicating loss of photoreceptors (Fig. 2F, 2I).

GFAP immunostaining was performed to assess Müller glia activation in response to retinal injury and degeneration. GFAP-positive staining was mainly located in the retinal surface (inner limiting membrane) and in donor cells delivered at early and later time points (Fig. 2J, 2K, 2M, 2N). In contrast, untreated or BSS-treated retinas showed more pronounced Müller glial activation with GFAP-positive inner and outer limiting membranes (Fig. 2L, 2O). More reactive Müller glial cells were also present in the retina after intervention at the later time point (Fig. 2M, 2N) compared with the early time point (Fig. 2J, 2K). In addition to activated Müller glia, grafted hNPC and hNPC^GDNF^ also express GFAP in the subretinal space (Fig. 2J, 2K, 2M, 2N), which is in concordance with our published reports.^35,41–43^

### hNPC and hNPC^GDNF^ Survive and Migrate in the Subretinal Space with Limited Proliferation

**Figure 3.**
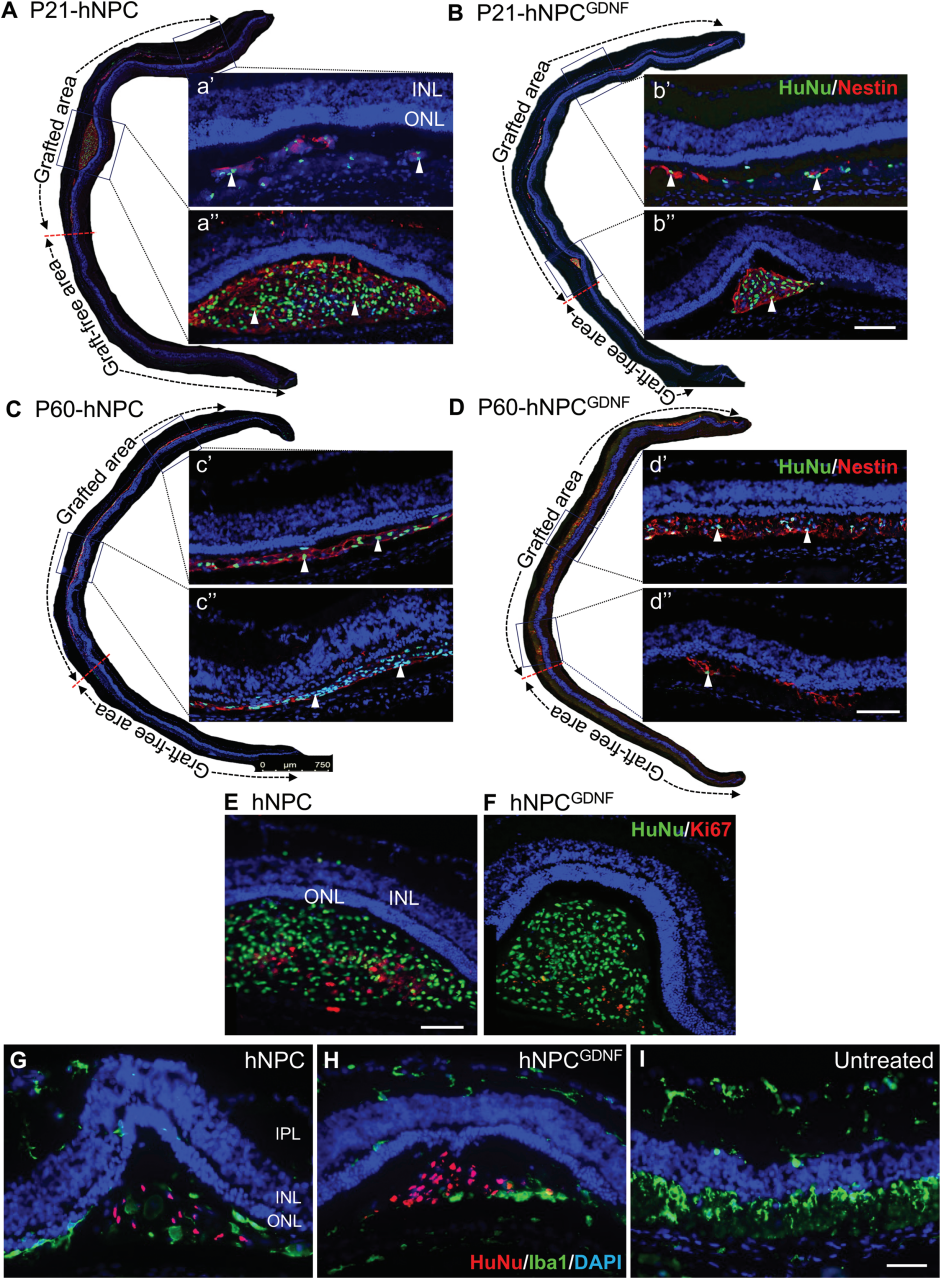
hNPC and hNPC^GDNF^ survive, migrate, and suppress microglial activation in the degenerative retinal environment. **(A-D)** Double immunostaining of human-specific markers (nuclear marker, HuNu, triangles and neural progenitor marker, Nestin) show survival and migration of hNPC and hNPC^GDNF^ after injection at P21 **(A&B)** or P60 **(C&D)**. aʹ-d″ are high-power images of the corresponding outlines. Graft-protected and graft-free areas are demarcated by bi-headed dotted arrows. **(E-F)** Double immunostaining of human-specific nuclear marker (HuNu) with cell proliferation marker (Ki6) revealed very limited proliferation of transplanted hNPCs and hNPC^GDNF^ (arrow in F) in the subretinal space and there was no evidence of outgrowth or tumor formation. **(G-I)** Immunostaining of microglia marker Iba1 ( with HuNu revealed reduced number of Iba1^+^ microglia in grafted areas of hNPC- and hNPC^GDNF^-treated retinas compared to untreated retina which showed a higher number of Iba1^+^ microglia. Scale bar = 750 μm for retinal montage images and 100 μm for high-power images. Abbreviations: INL, inner nuclear layer; ONL, outer nuclear layer; IPL, inner plexiform layer.

Immunofluorescence using human-specific antibodies for nuclear marker (HuNu) and neural progenitor marker (Nestin) confirmed hNPC and hNPC^GDNF^ survival and expression of Nestin following subretinal delivery at P21 or P60 (Fig. 3A–3D). Following injection at P21 or P60, hNPC and hNPC^GDNF^ showed similar distribution patterns either as a continuous line within the subretinal space between the RPE and photoreceptors or clumps of cells. Graft distribution correlated with extensive photoreceptor preservation up to 6-8 cell-thickness when intervention was at P21. Photoreceptor thickness of 3-4 layers remained unchanged with intervention at P60. Further, staining with the cell proliferation marker Ki67 and HuNu showed that hNPC or hNPC^GDNF^ grafts, especially in regions of clumps of cells, had very few double-positive cells (Fig. 3E, 3F). This finding is in concordance with our previous studies reporting that hNPC express low levels of telomerase in vitro and show diminishing cell proliferation in vivo.^42,44^ Staining for Ki67 alone likely indicates proliferating host microglia, as microglia activation is well-established in the RCS rat model.^45^

Pathological infiltration and activation of microglia have been well-established in the RCS rat model^45^ due to the accumulation of POS debris in the subretinal space. We sought to examine the status of microglial infiltration and activation in the grafted site of hNPC- and hNPC^GDNF^-treated retina by performing immunostaining of ionized calcium-binding adaptor molecule-1 (Iba1), in the lump of grafted human cells (HuNu) and untreated retina. Double immunostaining shows reduced numbers of Iba1^+^ microglia in the grafted area of the hNPC- and hNPC^GDNF^-treated retinas compared to the untreated retina that showed higher numbers of Iba1^+^ microglia in close vicinity to the degenerative photoreceptor layer (Fig. 3G–3I).

### hNPC^GDNF^ Produces GDNF In Vivo

**Figure 4.**
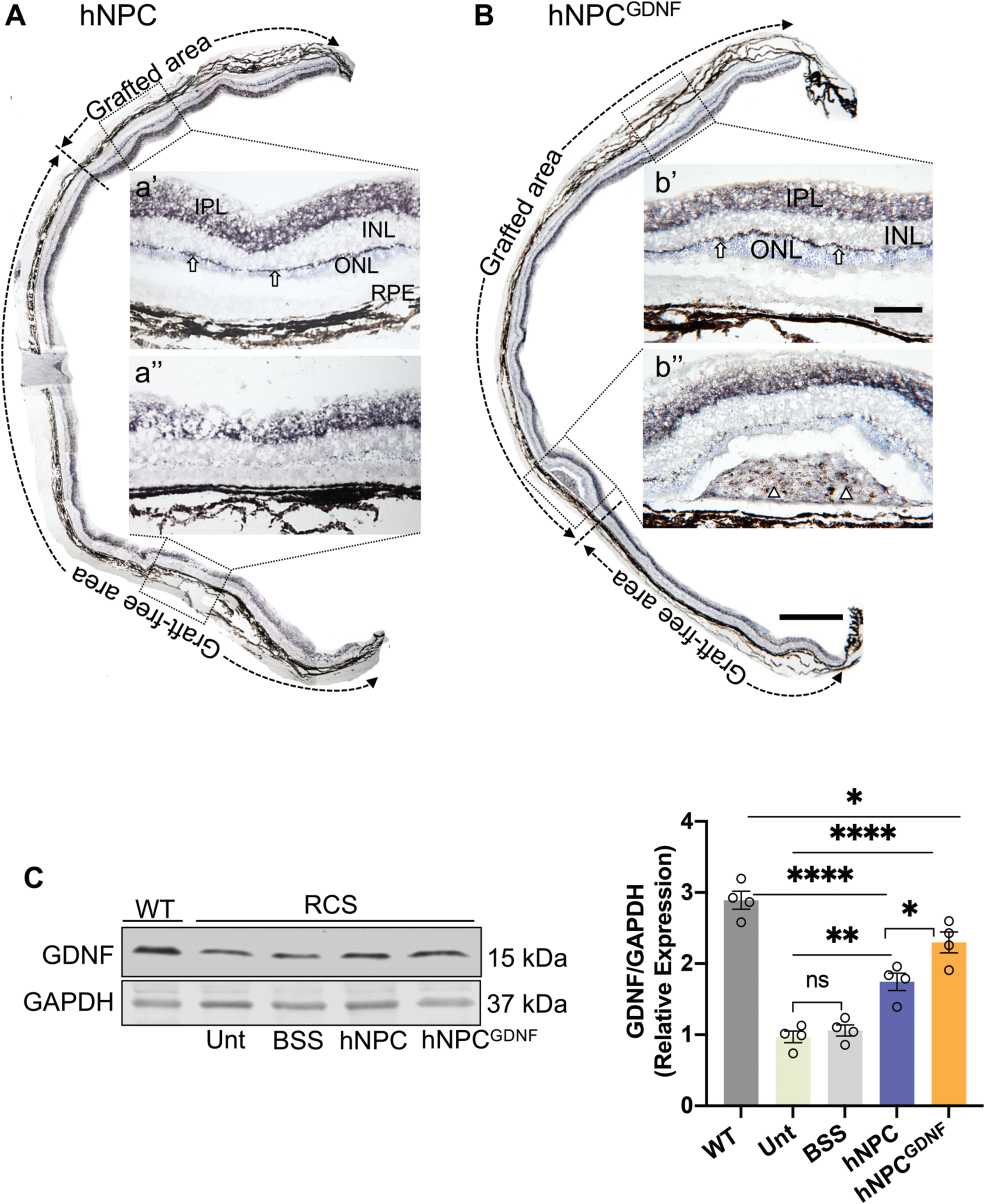
hNPC^GDNF^ produces GDNF in RCS rat retina. **(A-B)** Immunostaining of GDNF shows endogenous GDNF in OPL and IPL in both hNPC **(A)** and hNPC^GDNF^-treated **(B)** retina. Stronger GDNF staining was seen in the grafted area compared with area distant from the graft (aʹ vs. a″). aʹ& a″ and bʹ & b″ are high-power images from the corresponding outlines. Note, positive immunostaining of GDNF within the clump (b**″**) and individual (triangles in bʹ) of transplanted hNPC^GDNF^. Scale bar = 750 μm for montage images and 100 μm for high-power images. INL, inner nuclear layer; ONL, outer nuclear layer; IPL, inner plexiform layer. Graft-protected and graft-free areas are demarcated by bi-headed dotted arrows **(C)** Representative immunoblot and densitometric analysis shows significantly decreased GDNF production in all groups of RCS rats compared to the wild-type rat retina. GDNF production was significantly increased in the cell-treated retina compared to untreated and BSS retina, and GDNF production was significantly increased in the retina treated with hNPC^GDNF^ compared to hNPC. WT, wild-type, Unt, untreated; BSS, balanced salt solution. Data are represented as mean ± SEM. One-way ANOVA with Tukey’s test was used for multiple comparisons. *P ≤ .05, **P ≤ .01, ****P ≤ .0001, ns: nonsignificant.

As expected, positive GDNF immunostaining was detected from host cells in the inner plexiform layer (IPL), outer plexiform layer (OPL), and Müller cells (Fig. 4A, 4B). Critically, grafted hNPC^GDNF^ also show GDNF staining (lump of cells in Fig. 4b″) as well as in migrating cells (Fig. 4bʹ, high magnification, arrowheads), up to 70 days post-delivery.

Western blot analysis demonstrated significantly decreased GDNF expression in RCS retinas compared to wild-type retinas (Fig. 4C). hNPC treatment led to significantly increased GDNF levels compared to controls, presumably due to the protection of host GDNF-positive cells. As expected, hNPC^GDNF^-treated retinas showed significantly increased GDNF levels compared to controls, as well as increased GDNF levels compared to hNPC treatment. While hNPC and hNPC^GDNF^ treatment led to significantly increased GDNF levels compared to RCS rats, the level remained significantly lower than in the wild-type retina.

### hNPC and hNPC^GDNF^ Activate Various Cell Survival Pathways

**Figure 5.**
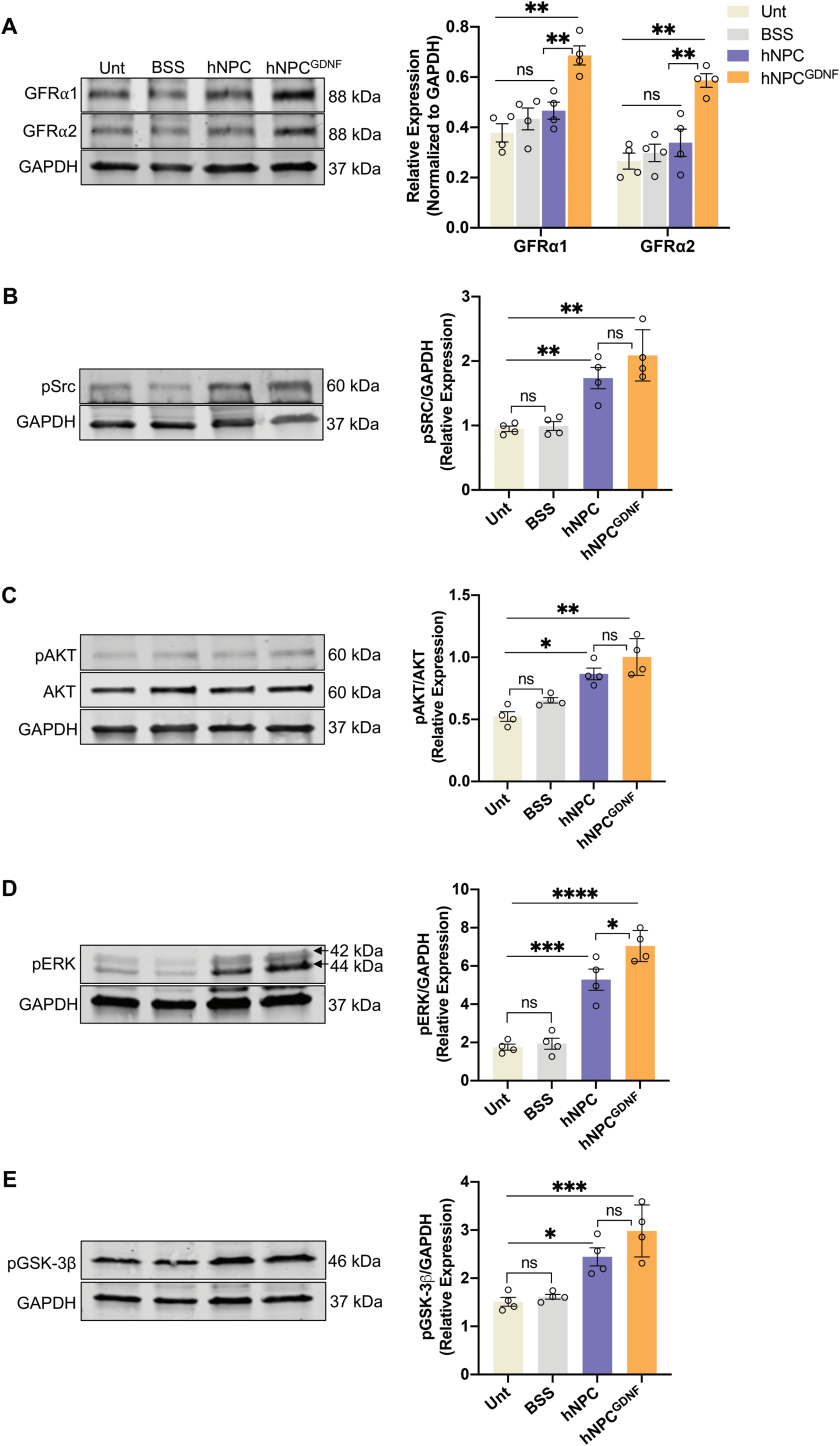
hNPC and hNPC^GDNF^ activate cell survival mediators in the RCS rat retina. **(A)** Representative immunoblots and densitometric analysis show significantly increased GFRα1 and GFRα2 expression in hNPC^GDNF^-treated retina compared to hNPC-treated, untreated, and BSS retina. **(B-E)** Representative immunoblots and densitometric analysis show significantly increased levels of the pSrc **(B)**, pAKT **(C)**, pERK **(D)**, and pGSK-3β **(E)** in retinas treated with hNPC- and hNPC^GDNF^ compared to the untreated and BSS retina. pERK was significantly increased in the hNPC^GDNF^-treated compared to hNPC. BSS, balanced salt solution; Unt, untreated. Data represented as mean ± SEM (*n* = 4). One-way ANOVA with Tukey’s test was used for multiple comparisons. *P ≤ .05, **P ≤ .01, ***P ≤ .001, ****P ≤ .0001; ns: non-significant.

We have previously shown that hNPC endogenously secrete neurotrophic factors such as FGF-2 and IGF-1 and further demonstrated that hNPC^GDNF^ enhances retinal protection compared to hNPC alone.^33^ Growth factors interact with their receptors, activate different cell survival pathways, and induce nuclear factor erythroid 2-related factor 2 (Nrf2)-dependent antioxidant responses to exert neuroprotective effects.^46–50^ In particular, GDNF is reported to act through RET/GFRα1 or 2 to activate various cell survival pathways, which may phosphorylate Nrf2 and activate downstream signaling cascades.^51–55^ Immunoblot analysis revealed significantly increased expression of GFRα1 and GFRα2 in hNPC^GDNF^-treated retinas compared to other groups (*P* < .01, Fig. 5A). Compared to hNPC alone, significantly increased expression of GFRα1 and GFRα2 only in hNPC^GDNF^-treated retinas strongly support that the enhanced protective role of hNPC^GDNF^ is mediated by GDNF-dependent mechanism(s). Hence, we evaluated the expression levels of phosphorylated forms of the most common cell-survival signaling mediators downstream to GDNF, specifically Src, AKT, ERK, and GSK-3β in the retinas of all the groups. Immunoblot analysis demonstrated that levels of pSrc (Fig. 5B), pAKT (Fig. 5C), pERK (Fig. 5D), and pGSK-3β (Fig. 5E) were significantly increased in both hNPC- and hNPC^GDNF^-treated retinas compared to control retinas. Additionally, hNPC^GDNF^-treated compared to hNPC-treated retinas showed a significant difference in the levels of pERK. Taken together, these data suggest that increased phosphorylation of Src, AKT, GSK-3β, and ERK promote photoreceptor survival, maybe through activating cell survival signaling cascades and/or Nrf2-dependent antioxidant responses.

### hNPC and hNPC^GDNF^ Reduce Oxidative Stress

**Figure 6.**
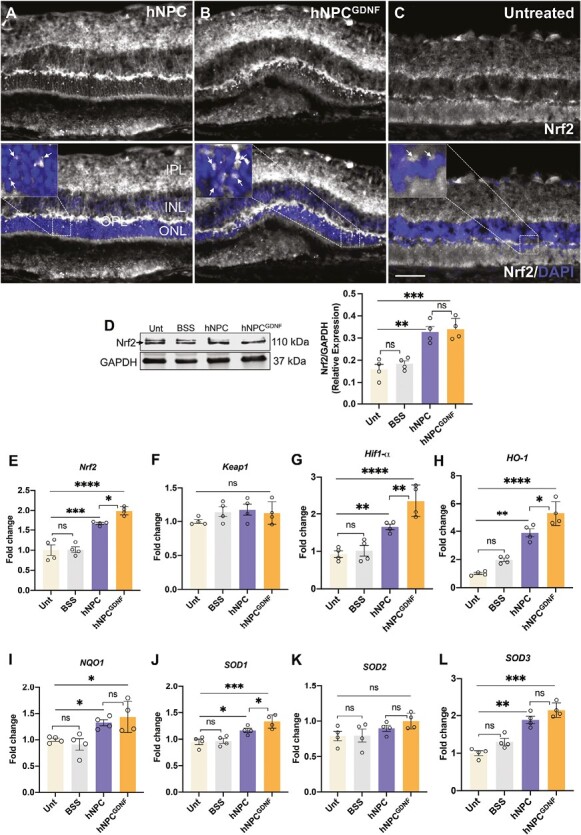
hNPC and hNPC^GDNF^ reduce oxidative stress in RCS rat retina. **(A-C)** Immunostaining of Nrf2 showed intense immunoreactivity in OPL, IPL, and RGCs of both hNPC- **(A)** and hNPC^GDNF^-treated **(B)** retina compared to untreated retina **(C)**. Higher Nrf2 puncta and its significantly increased nuclear localization (inset: white arrows) were observed in hNPC^GDNF^-treated compared to hNPC-treated retina. Scale bar = 100 μm. INL, inner nuclear layer; ONL, outer nuclear layer; OPL, outer plexiform layer; INL, inner nuclear layer; IPL, inner plexiform layer. **(D)** Representative immunoblot and densitometric analysis showed significantly increased Nrf2 levels in both hNPC- and hNPC^GDNF^-treated retina compared to untreated and BSS retina. **(E-L)** qRT-PCR demonstrated that *Nrf2, Hif1-α***,***HO-1, NQO1, SOD1,* and *SOD3* mRNA transcripts were significantly increased in the hNPC- and hNPC^GDNF^-treated retina compared to the untreated and BSS retina. *Nrf2, Hif1-α, HO-1, SOD1* mRNA transcripts were significantly increased in retinas treated with hNPC^GDNF^ compared to hNPC. No change in *Keap1* and *SOD2* expression was observed across groups. Unt, untreated; BSS, balanced salt solution. Data are represented as mean ± SEM (*n* = 4). One-way ANOVA with Tukey’s test was used for multiple comparisons. *P ≤ .05, **P ≤ .01, ***P ≤ .001, ****P ≤ .0001, ns: nonsignificant.

Oxidative stress plays a key role in retinal degeneration.^56–58^ Nrf2 is a redox-sensitive transcription factor that governs cellular defense mechanisms against oxidative stress. Upon activation, Nrf2 is released from its cytoplasmic-binding partner Keap1 and translocated to the nucleus where it activates antioxidant enzymes such as heme oxygenase-1 (HO-1) and NAD(P) H:quinone oxidoreductase 1 (NQO1), thereby delaying the progression of RP.^59–62^ We next sought to investigate whether hNPC and hNPC^GDNF^ treatment alter oxidative stress regulatory transcription factors (Nrf2) and hypoxia-inducible factor 1-alpha (Hif1α) and the expression of antioxidant enzymes (HO-1, NQO1, and superoxide dismutases: SOD1, SOD2, SOD3) to reduce oxidative stress. Intense immunoreactivity of Nrf2 was documented in the OPL, IPL, and RGC layer of both hNPC- and hNPC^GDNF^-treated retina compared to untreated retina (Fig. 6A–6C), suggesting the activation of Nrf2 and its downstream signaling against oxidative stress. Nuclear localization of Nrf2 (Fig. 6A, 6B, inset: white arrows) was observed in the ONL of grafted areas of both hNPC- and hNPC^GDNF^-treated retina. On the other hand, no nuclear Nrf2 was observed in untreated retina. Immunoblot and corresponding densitometric analysis confirm significantly increased Nrf2 levels in hNPC- and hNPC^GDNF^-treated retina compared to BSS and untreated retina (Fig. 6D). Evaluation of the mRNA transcripts showed significantly increased expression of *Nrf2* and *Hif1α* in hNPC- and hNPC^GDNF^-treated retinas compared to control retinas, while *Keap1* levels were similar between groups (Fig. 6E–6G). Expression of all assessed antioxidant enzymes, except for SOD2, was upregulated in both cell-treated retinas compared to control retinas (Fig. 6H–6M). Moreover, upregulation of *Nrf2, Hif1α, HO-1,* and *SOD1* was significantly more pronounced in hNPC^GDNF^-treated than in hNPC-treated retinas.

### hNPC and hNPC^GDNF^ Upregulate Markers of Phagocytosis and Autophagy

Homeostasis of shedding POS and their phagocytic degradation by RPE maintains normal vision.^27,63,64^ In RPE, the phagocytic degradation of POS is mediated by a noncanonical form of autophagy mechanism known as LC3-associated phagocytosis (LAP).^65,66^ Disruption of RPE phagocytosis leads to degeneration of photoreceptors and other retinal cells over time. Our previous studies demonstrated that NPC provide photoreceptor protection by eliminating POS through phagocytosis^39^; however, the underlying mechanism(s) are yet to be elucidated. In general, phagocytic cells are shown to maintain tissue homeostasis by forming phagolysosomes and eliminating senescent cells, cell fragments, and apoptotic bodies.^67–70^ Phagolysosome formation requires lysosomal-associated membrane protein 1 (LAMP1) which mediates the fusion of lysosomes with phagosomes.^67,71^ A hallmark of degradative lysosomes is the presence of activated lysosomal proteolytic enzymes including cathepsins that mediate POS degradation in the phagolysosome. Cathepsin D (CTSD) is one of the major active lysosomal aspartyl proteases present in the degradative lysosomes.^72^ Furthermore, the interactions and co-partnering of LAMP1 with CTSD promote the formation of degradative lysosomes to mediate POS degradation.^73^

**Figure 7.**
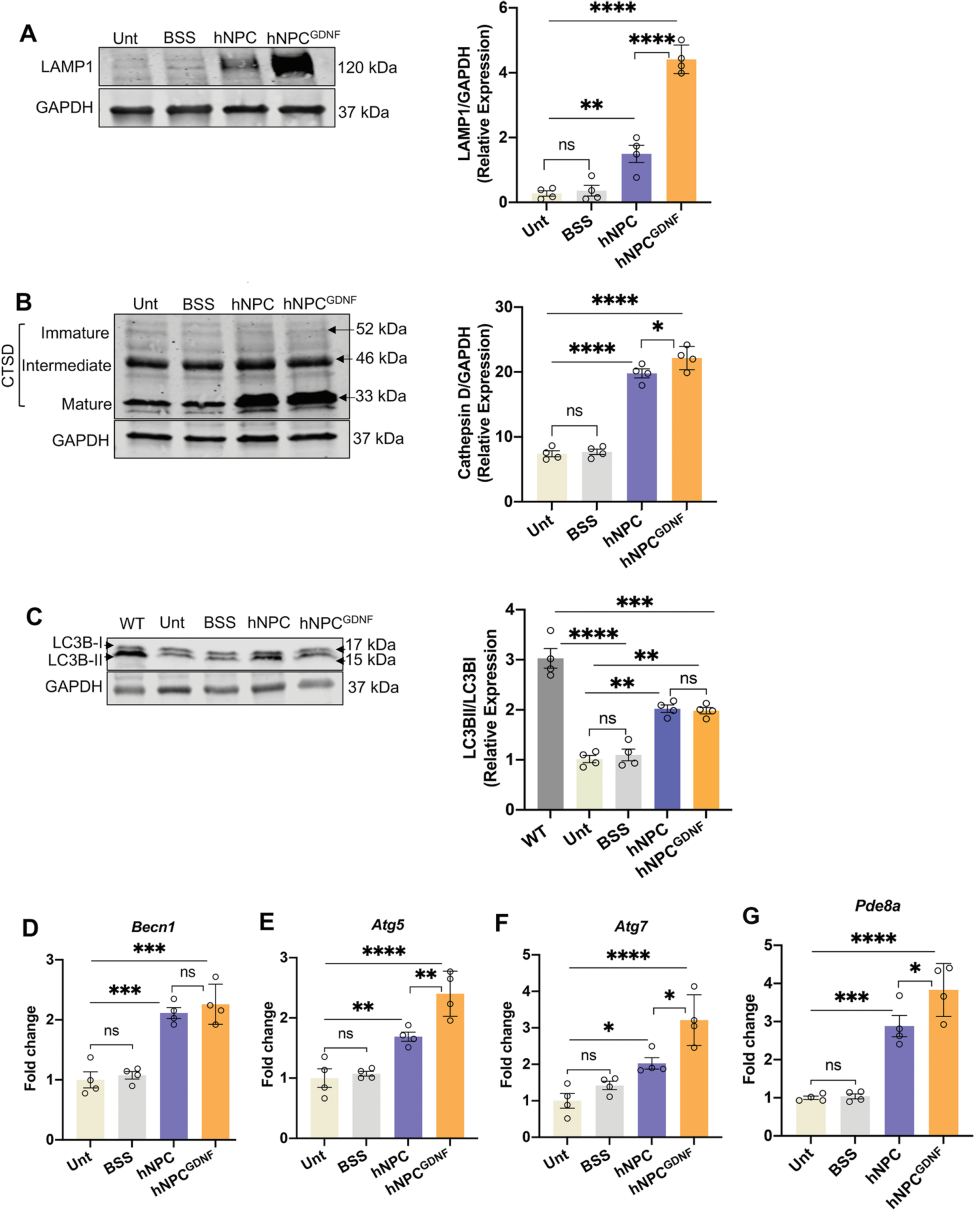
hNPC and hNPC^GDNF^ mediate phagolysosomal degradation of POS and restore autophagy in RCS rat retina. **(A-C)** Representative immunoblots and densitometric analysis showed significantly increased Cathepsin D **(A)** and LAMP1 **(B)** levels in both hNPC- and hNPC^GDNF^-treated retina, compared to controls. LAMP1 levels were significantly increased in retinas treated with hNPC^GDNF^ compared to hNPC. Decreased levels of LC3B-II (lipidated form of LC3B) occurred in untreated, BSS-, hNPC-, and hNPC^GDNF^-treated RCS compared to the wild-type retina **(C)**. LC3B-II levels were significantly increased in the hNPC- and hNPC^GDNF^-treated retinas compared to the untreated and BSS retinas, but not to wild-type levels. **(D-G)** qPCR shows that *Becn1***(D)**, *Atg5***(E)**, *Atg7***(F)**, and *Pde8a***(G)** mRNA transcripts were significantly increased in hNPC- and hNPC^GDNF^-treated retinas compared to the untreated and BSS retina. *Atg5*, *Atg7*, and *Pde8a* mRNA transcripts were significantly increased in retinas treated with hNPC^GDNF^ compared to hNPC. BSS, balanced salt solution; Unt, untreated; WT, wild-type. Data represented as mean ± SEM (*n* = 4). One-way ANOVA with Tukey’s test was used for multiple comparisons. *P ≤ .05, **P ≤ .01, ***P ≤ .001; ****P ≤ .0001; ns: non-significant.

Western blot analysis showed that LAMP1 and CTSD (mature form: ~33 kDa) levels were significantly increased in both hNPC and hNPC^GDNF^-treated retinas compared to untreated and BSS retina (Fig. 7A, 7B). Critically, LAMP1 and CTSD levels were significantly increased following hNPC^GDNF^ treatment compared to hNPC. No change was observed in the immature (~52 kDa) and intermediate (~46 kDa) forms of CTSD in hNPC and hNPC^GDNF^-treated retinas compared to controls. In addition, triple immunostaining using RPE cell marker (RPE65), Nestin and CTSD demonstrated preservation of the RPE with normal morphology in the grafted area of hNPC and hNPC^GDNF^-treated retinas, similar to wild-type control. Further, intense immunoreactivity and higher puncta of CTSD in the RPE layer of hNPC and hNPC^GDNF^-treated retinas, similar to WT control (Supplementary Fig. S4A) may suggest the restoration of phagocytic activity of RPE in the hNPC and hNPC^GDNF^-treated retinas. Furthermore, double immunostaining of CTSD (green) with HuNu (red) showed intense peri- and intra-nuclear immunoreactivity of CTSD and its colocalization with HuNu in the transplanted hNPC and hNPC^GDNF^(Supplementary Fig. S4B) may also suggest the phagocytic activity of hNPC and hNPC^GDNF^. In untreated retinas, CTSD puncta were observed in both the RPE and ONL around the nuclei of degenerating photoreceptors; yet no immunoreactivity was detected in the ONL of hNPC and hNPC^GDNF^-treated retinas.

Autophagy plays a critical role in POS phagocytosis and maintaining photoreceptor health.^65,66,74,75^ Studies have reported lysosomal membrane permeabilization and autophagy blockade as key contributors to photoreceptor death.^74,76–78^ In conventional autophagy, the lipidated form of LC3B (LC3B-II) is associated with the double-membrane autophagosomes. The LC3B-II/ LC3B-I ratio serves as a key marker of autophagic flux^79^ and, importantly, this ratio plays a key role in maintaining autophagy homeostasis.^80^ Investigating markers of autophagy following cell treatment showed that the LC3B-II and the LC3B-II/LC3B-I ratio were significantly decreased in the untreated and BSS RCS retinas compared to wild-type and that both were significantly increased in hNPC- and hNPC^GDNF^-treated retinas compared to the untreated and BSS retinas (Fig. 7C). Furthermore, expression of key autophagic genes, *Becn1, Atg5,* and *Atg7,* was also increased in hNPC- and hNPC^GDNF^-treated retinas compared to controls (Fig. 7D–7F). Notably, both *Atg5* and *Atg7* expression was significantly higher with hNPC^GDNF^ treatment compared to hNPC. Collectively, increased phagocytosis and autophagy could play a role in preserved vision with hNPC- and hNPC^GDNF^ and these may be augmented with hNPC^GDNF^ treatment. Based likely on multiple modes of action, retinas treated with hNPC or hNPC^GDNF^ compared to controls had significantly increased expression of *Pde8a*, one of the key photoreceptor phototransduction genes, and this was further augmented with hNPC^GDNF^ treatment compared to hNPC alone (Fig. 7G).

## Discussion

Positive results from preclinical studies using stem cells to treat retinal degenerative diseases have often not been reflected in subsequent clinical trials. This may largely reflect that most preclinical studies were conducted with animal models at an early stage of disease. This includes our studies at an early stage of disease intervention, showing that hNPC can offer vision protection and that this is enhanced with hNPC^GDNF^.^33^ Given that RP patients selected for clinical trials and presented to the clinic have more advanced retinal degeneration, testing stem cell therapy in later-stage animal models is clinically relevant. This study showed that both hNPC and hNPC^GDNF^ offer dramatic vision and retinal preservation. Protection was provided by treatment even at later stages of retinal degeneration. hNPC^GDNF^ compared with hNPC treatment provided significantly enhanced visual function when intervention was at the early stage of disease and offered significantly broader photoreceptor protection at both early and late intervention times.

GFAP immunoreactivity was observed in the grafted hNPC and hNPC^GDNF^ in the subretinal space and is in concordance with our previous studies in animals^35,41,42^ and most recently in the human spinal cord.^43^ The neural progenitors are not terminally differentiated before transplantation, and GFAP expression in grafted hNPC and hNPC^GDNF^ indicates their potential to differentiate into astrocytes in vivo. Very few grafted cells were labeled with the proliferation marker Ki67, which is in concordance with our previous demonstrations that hNPC express low levels of telomerase in vitro and show diminishing cell proliferation over time in vivo.^42,44^ This finding indicates that hNPC or hNPC^GDNF^ are likely non-tumorigenic, which we have reported in a long-term study in the rodent subretinal space and recently showed in the human spinal cord up to 42 months post-transplantation.^43,81^

Visual acuity tested by OKR revealed that hNPC and hNPC^GDNF^ delivered at early and late time points provided similar protection. This is likely because OKR already reached nearly normal levels with hNPC alone, and therefore no further protection was detected by hNPC^GDNF^. Cell treatment maintained visual acuity over time in contrast to control groups with clear deterioration from P60 to P90. OKR only needs local photoreceptors at the dorsal-temporal part of the retina to record a response and, hence, local versus extensive photoreceptor preservation is not distinguished. In contrast, ERG measures the average of whole retinal activities to light stimulation and requires a larger photoreceptor area to have a recordable response. Delivering both hNPC and hNPC^GDNF^ at the early stage of degeneration led to significantly higher b-wave amplitude compared with controls. Critically, hNPC^GDNF^ treatment provided significantly higher b-wave amplitude, as well as photoreceptor coverage, compared to hNPC. In contrast, neither cell type preserved b-wave amplitude at the later treatment time point, which may be due to several following factors.

An elegant study revealed that long-term vision protection with RPE65 gene therapy may only occur in retinal regions with larger than 63% retained photoreceptors at the time of intervention.^82^ While donor cells migrate extensively in the subretinal space, even with the later delivery time point, a single subretinal injection affects only approximately 1/3 of the retinal area. Therefore, the majority of the retina distal to grafted cells still undergoes progressive degeneration, which we demonstrated with luminance thresholds that remain unchanged within the central graft but were elevated at the periphery.^81^ With P60 intervention, it is likely that local preserved photoreceptors can produce an OKR, but not a recordable ERG requiring a broader area of functional photoreceptors. In addition, inflammation, oxidative stress, and trophic factor deprivation increase with progressive retinal degeneration.^83–86^ This hostile environment may reduce the effectiveness of donor cells at the periphery of the graft site. Future studies can investigate top-regulated genes/proteins that change over time in donor cells and the host retina to optimize long-term vision protection.

Though broad visual function based on ERG was not recorded with later treatment, photoreceptor coverage and expression of a key photoreceptor phototransduction gene were significantly greater with hNPC^GDNF^ treatment compared to hNPC, suggesting that photoreceptor protection benefits from more than stem cells alone. We have previously demonstrated that hNPC can regulate immune response by inhibiting microglia activation, promote an antioxidant effect by upregulating Nrf2, clear POS by phagocytosis^39,87^ and provide neuroprotective effects by releasing trophic factors FGF-2 and IGF-1.^33^ Several of these mechanisms appear to be enhanced with hNPC^GDNF^ compared to hNPC alone, which may underlie the increased protective effects. First, sustained GDNF release can directly protect photoreceptors via their receptors and indirectly protect photoreceptors via activation of retinal Müller glia, which then increase the production of various growth factors. While there was a reactive Müller glial response in the degenerative retina, this endogenous GDNF was not sufficient to rescue the photoreceptor from degeneration. This is the first study to show exogenous GDNF in the retina from hNPC^GDNF^ both within the graft core and migrating out. Extensive GDNF release is likely central to the protective effects of hNPC^GDNF^ in the RCS rat. GDNF binds to the GDNF-family receptor complex (GFRα1/α2-RET), leading to phosphorylation and activation of Src-, AKT-, GSK3β-, and ERK-dependent signaling to protect neurons from degeneration and support their survival.^88,89^ Retinas treated with hNPC^GDNF^, compared to hNPC alone, showed higher levels of GFRα1/α2-RET.

Retinas treated with hNPC^GDNF^ also had higher levels of pERK, compared to hNPC alone. While the exact mechanism for enhanced vision protection with hNPC^GDNF^ compared to hNPC is not entirely clear, it is likely to involve activation of ERK as a key survival pathway. In addition, increased phosphorylation of AKT, GSK3β, and ERK has been shown to regulate the phagocytic activity of RPE POS debris.^73,90^ Here, genes and proteins related to phagocytosis and degradation of POS were significantly upregulated in hNPC^GDNF^-treated retinas compared to other groups, indicating that removal of POS contributes to enhanced vision protection.

We have previously demonstrated that transplanted human neural stem cells and iPSC-derived NPC can phagocytose the POS in the RCS retina.^39,91^ In addition to their own phagocytic activity, hNPC or hNPC^GDNF^-treatment, through their secretory growth factor milieu, may also partially restore the phagocytic activity of host RPE cells. Moreover, stable release of GDNF from hNPC^GDNF^ may further supplement and restore some RPE function, compared to hNPC. This is similar to a report that human umbilical tissue-derived stem cells rescue RPE phagocytic function by secreting various growth factors including GDNF.^92^ Photoreceptor protection in the hNPC- or hNPC^GDNF^-treated RCS retina via elimination of accumulated POS, may result from phagocytic action of both grafted cells and partially restored host RPE. The phagocytic role of NPC appears to support RPE cell functions and the secreted GDNF offers neuroprotection and enables the extended survival of photoreceptor cells in transplanted animal eyes. However, further studies are required to delineate the role of partial functional restoration of RPE following hNPC- or hNPC^GDNF^ treatment.

Based on the activation of several key genes, such as *Nrf2*, it appears that grafted hNPC^GDNF^ may exert a protective effect through a reduction in oxidative stress. Under physiological conditions, the retina is characterized by a high oxygen consumption rate and intense exposure to light. Therefore, the retina is very susceptible to oxidative stress, with studies showing that oxidative stress plays a prominent role in the pathogenesis of degenerative retinal diseases such as age-related macular degeneration (AMD), glaucoma, diabetic retinopathy, and RP.^84,86,93–95^ In fact, therapeutic interventions that reduce oxidative stress can delay retinal degeneration.^96,97^ Therefore, an ability of hNPC^GDNF^ to reduce oxidative stress may contribute to the enhanced effect over hNPC alone.

This initial study used only the one late time point of P60 delivery, yet multiple time points should be tested to optimize the delivery window for stem cell and growth factor treatment. Additionally, while GDNF is clearly protective, GDNF has been reported to cause aberrant sprouting and negative feedback effects on neurotransmitter homeostasis^88,98,99^ and high doses of GDNF plasmid led to a significant reduction of scotopic b-wave amplitude and photoreceptor thickness.^100^ A long-term tumor/ toxicology study is next required to determine long-term cell function and toxicity. However, numerous previous studies delivering hNPC^GDNF^ both in the central nervous system (CNS) and eyes are encouraging, as neuroprotection was provided without observed side effects. Finally, this and prior studies confirm limited donor cell proliferation, which is critical for translation to a human trial. Based on the effectiveness of hNPC^GDNF^ for retinal protection following delivery at a clinically relevant stage of disease, these cells should next be assessed in IND-enabling studies for translation to the clinic.

### Potential Clinical Application

Our extensive preclinical studies have shown that hNPC provides a promising treatment for RP, and this led to an ongoing phase I/2a clinical trial delivering the clinical product, CNS10-NPC, to patients with RP (NCT04284293). Under GMP, we have genetically engineered CNS10-NPC to stably secrete GDNF, and expanded and banked this cell line (termed, CNS10-NPC-GDNF) as a clinical product.^36^ CNS10-NPC-GDNF has recently been delivered to the lumbar spinal cord of 18 patients with amyotrophic lateral sclerosis (ALS) in a phase I/2a clinical trial (NCT02943850), which met the primary endpoint of safety.^43^ We now show that combining hNPC with GDNF may be even more effective than hNPC alone to treat RP. In addition, a recent study showed that RPE phagocytosis significantly declined in AMD) compared with only a moderate decline in controls. Remarkably, GDNF increases AMD RPE phagocytosis,^101^ indicating hNPC^GDNF^ has the potential to treat retinal degeneration including both RP and AMD. Unlike the RPE cells which need Brunch’s membrane for survival and function, hNPC^GDNF^ survive and migrate long distances in the subretinal space, release multiple growth factors for photoreceptor protection, enhance RPE phagocytosis and mediate various survival pathways. Given the encouraging preclinical outcomes with hNPC^GDNF^ shown here, as well as long-term safety of CNS10-NPC-GDNF in the ALS spinal cord, this cell product holds great promise as a therapeutic option for RP and other retinal diseases.

## Supplementary Material

szad054_suppl_Supplementary_Figures

szad054_suppl_Supplementary_Table

## Data Availability

Data that support the findings of this study are available from the corresponding authors upon reasonable request.

## References

[1] Lange R , Kumagai A, Weiss S, et al. Vision-related quality of life in adults with severe peripheral vision loss: a qualitative interview study. J Patient Rep Outcomes. 2021;5(1):7. 10.1186/s41687-020-00281-y 33439361 PMC7806695

[2] Enoch J , McDonald L, Jones L, Jones PR, Crabb DP. Evaluating whether sight is the most valued sense. JAMA Ophthalmol. 2019;137(11):1317-1320. 10.1001/jamaophthalmol.2019.3537 31580383 PMC6777262

[3] Berson EL. Retinitis pigmentosa. The friedenwald lecture. Invest Ophthalmol Vis Sci. 1993;34(5):1659-1676. 8473105

[4] Hartong DT , Berson EL, Dryja TP. Retinitis pigmentosa. Lancet. 2006;368(9549):1795-1809. 10.1016/S0140-6736(06)69740-7 17113430

[5] Wang AL , Knight DK, Vu TT, Mehta MC. Retinitis pigmentosa: review of current treatment. Int Ophthalmol Clin. 2019;59(1):263-280. 10.1097/IIO.0000000000000256 30585930

[6] Fahim A. Retinitis pigmentosa: recent advances and future directions in diagnosis and management. Curr Opin Pediatr. 2018;30(6):725-733. 10.1097/MOP.0000000000000690 30234647

[7] Botto C , Rucli M, Tekinsoy MD, et al. Early and late stage gene therapy interventions for inherited retinal degenerations. Prog Retin Eye Res. 2022;86:100975. 10.1016/j.preteyeres.2021.100975 34058340

[8] Liu W , Liu S, Li P, et al. Retinitis pigmentosa: progress in molecular pathology and biotherapeutical strategies. Int J Mol Sci. 2022;23(9). 10.3390/ijms23094883 PMC9101511 35563274

[9] Miraldi Utz V , Coussa RG, Antaki F, Traboulsi EI. Gene therapy for RPE65-related retinal disease. Ophthalmic Genet. 2018;39(6):671-677. 10.1080/13816810.2018.1533027 30335549

[10] Cideciyan AV , Jacobson SG, Beltran WA, et al. Human retinal gene therapy for Leber congenital amaurosis shows advancing retinal degeneration despite enduring visual improvement. Proc Natl Acad Sci USA. 2013;110(6):E517-E525. 10.1073/pnas.1218933110 23341635 PMC3568385

[11] Gao J , Hussain RM, Weng CY. Voretigene neparvovec in retinal diseases: a review of the current clinical evidence. Clin Ophthalmol. 2020;14:3855-3869. 10.2147/OPTH.S231804 33223822 PMC7671481

[12] Deng C , Zhao PY, Branham K, et al. Real-world outcomes of voretigene neparvovec treatment in pediatric patients with RPE65-associated Leber congenital amaurosis. Graefes Arch Clin Exp Ophthalmol. 2022;260(5):1543-1550. 10.1007/s00417-021-05508-2 35001204 PMC9010358

[13] Jones MK , Lu B, Girman S, Wang S. Cell-based therapeutic strategies for replacement and preservation in retinal degenerative diseases. Prog Retin Eye Res. 2017;58:1-27. 10.1016/j.preteyeres.2017.01.004 28111323 PMC5441967

[14] Zarbin M. Cell-based therapy for degenerative retinal disease. Trends Mol Med. 2016;22(2):115-134. 10.1016/j.molmed.2015.12.007 26791247

[15] Singh MS , Park SS, Albini TA, et al. Retinal stem cell transplantation: balancing safety and potential. Prog Retin Eye Res. 2020;75:100779. 10.1016/j.preteyeres.2019.100779 31494256 PMC7056514

[16] Zhang K , Hopkins JJ, Heier JS, et al. Ciliary neurotrophic factor delivered by encapsulated cell intraocular implants for treatment of geographic atrophy in age-related macular degeneration. Proc Natl Acad Sci USA. 2011;108(15):6241-6245. 10.1073/pnas.1018987108 21444807 PMC3076847

[17] Yang JY , Lu B, Feng Q, et al. Retinal protection by sustained nanoparticle delivery of oncostatin M and ciliary neurotrophic factor into rodent models of retinal degeneration. Transl Vis Sci Technol. 2021;10(9):6. 10.1167/tvst.10.9.6 PMC8340648 34347033

[18] LaVail MM , Unoki K, Yasumura D, et al. Multiple growth factors, cytokines, and neurotrophins rescue photoreceptors from the damaging effects of constant light. Proc Natl Acad Sci USA. 1992;89(23):11249-11253. 10.1073/pnas.89.23.11249 1454803 PMC50527

[19] Di Pierdomenico J , Scholz R, Valiente-Soriano FJ, et al. Neuroprotective effects of FGF2 and minocycline in two animal models of inherited retinal degeneration. Invest Ophthalmol Vis Sci. 2018;59(11):4392-4403. 10.1167/iovs.18-24621 30193320

[20] Michelis G , German OL, Villasmil R, et al. Pigment epithelium-derived factor (PEDF) and derived peptides promote survival and differentiation of photoreceptors and induce neurite-outgrowth in amacrine neurons. J Neurochem. 2021;159(5):840-856. 10.1111/jnc.15454 34133756 PMC9590253

[21] Frasson M , Picaud S, Léveillard T, et al. Glial cell line-derived neurotrophic factor induces histologic and functional protection of rod photoreceptors in the rd/rd mouse. Invest Ophthalmol Vis Sci. 1999;40(11):2724-2734. 10509671

[22] Baranov P , Lin H, McCabe K, et al. A novel neuroprotective small molecule for glial cell derived neurotrophic factor induction and photoreceptor rescue. J Ocul Pharmacol Ther. 2017;33(5):412-422. 10.1089/jop.2016.0121 28441076 PMC5911694

[23] Delyfer MN , Simonutti M, Neveux N, Léveillard T, Sahel J-A. Does GDNF exert its neuroprotective effects on photoreceptors in the rd1 retina through the glial glutamate transporter GLAST? Mol Vis. 2005;11:677-687. 16163265

[24] Jomary C , Darrow RM, Wong P, Organisciak DT, Jones SE. Expression of neurturin, glial cell line-derived neurotrophic factor, and their receptor components in light-induced retinal degeneration. Invest Ophthalmol Vis Sci. 2004;45(4):1240-1246. 10.1167/iovs.03-1122 15037593

[25] Harada T , Harada C, Kohsaka S, et al. Microglia-Muller glia cell interactions control neurotrophic factor production during light-induced retinal degeneration. J Neurosci. 2002;22(21):9228-9236. 10.1523/JNEUROSCI.22-21-09228.2002 12417648 PMC6758038

[26] Dowling JE , Sidman RL. Inherited retinal dystrophy in the rat. J Cell Biol. 1962;14(1):73-109. 10.1083/jcb.14.1.73 13887627 PMC2106090

[27] D’Cruz PM , Yasumura D, Weir J, et al. Mutation of the receptor tyrosine kinase gene Mertk in the retinal dystrophic RCS rat. Hum Mol Genet. 2000;9(4):645-651. 10.1093/hmg/9.4.645 10699188

[28] LaVail MM. Fawn-hooded rats, the fawn mutation and interaction of pink-eyed and red-eyed dilution genes. J Hered. 1981;72(4):286-287. 10.1093/oxfordjournals.jhered.a109500 7288142

[29] Tzameret A , Sher I, Edelstain V, et al. Evaluation of visual function in Royal College of Surgeon rats using a depth perception visual cliff test. Vis Neurosci. 2019;36:E002. 10.1017/S095252381800007X 30700338

[30] Girman SV , Wang S, Lund RD. Time course of deterioration of rod and cone function in RCS rat and the effects of subretinal cell grafting: a light- and dark-adaptation study. Vision Res. 2005;45(3):343-354. 10.1016/j.visres.2004.08.023 15607350

[31] Sauve Y , Pinilla I, Lund RD. Partial preservation of rod and cone ERG function following subretinal injection of ARPE-19 cells in RCS rats. Vision Res. 2006;46(8-9):1459-1472. 10.1016/j.visres.2005.11.009 16364396

[32] Pinilla I , Lund RD, Sauve Y. Contribution of rod and cone pathways to the dark-adapted electroretinogram (ERG) b-wave following retinal degeneration in RCS rats. Vision Res. 2004;44(21):2467-2474. 10.1016/j.visres.2004.05.020 15358082

[33] Gamm DM , Wang S, Lu B, et al. Protection of visual functions by human neural progenitors in a rat model of retinal disease. PLoS One. 2007;2(3):e338. 10.1371/journal.pone.0000338 17396165 PMC1828619

[34] Lu B , Morgans CW, Girman S, et al. Neural stem cells derived by small molecules preserve vision. Transl Vis Sci Technol. 2013;2(1):1. 10.1167/tvst.2.1.1 PMC3763886 24049711

[35] Lu B , Tsai Y, Girman S, et al. A subsequent human neural progenitor transplant into the degenerate retina does not compromise initial graft survival or therapeutic efficacy. Trans Vis Sci Technol. 2015;4:1-14. 10.1167/tvst.4.1.7 PMC4324446 25694843

[36] Shelley BC , Gowing G, Svendsen CN. A cGMP-applicable expansion method for aggregates of human neural stem and progenitor cells derived from pluripotent stem cells or fetal brain tissue. J Vis Exp. 2014(88):51219. 10.3791/51219 24962813 PMC4195512

[37] Svendsen CN , ter Borg MG, Armstrong RJ, et al. A new method for the rapid and long term growth of human neural precursor cells. J Neurosci Methods. 1998;85(2):141-152. 10.1016/s0165-0270(98)00126-5 9874150

[38] Capowski EE , Schneider BL, Ebert AD, et al. Lentiviral vector-mediated genetic modification of human neural progenitor cells for ex vivo gene therapy. J Neurosci Methods. 2007;163(2):338-349. 10.1016/j.jneumeth.2007.02.022 17397931

[39] Tsai YC LB , Bakondi A, Girman S, et al. Human iPSC-derived neural progenitors preserve vision in an AMD-like model. Stem Cells. 2015;33(8):2537-2549. 25869002 10.1002/stem.2032 PMC5477659

[40] Douglas RM , Alam NM, Silver BD, et al. Independent visual threshold measurements in the two eyes of freely moving rats and mice using a virtual-reality optokinetic system. Vis Neurosci. 2005;22(5):677-684. 10.1017/S0952523805225166 16332278

[41] Das MM , Avalos P, Suezaki P, et al. Human neural progenitors differentiate into astrocytes and protect motor neurons in aging rats. Exp Neurol. 2016;280:41-49. 10.1016/j.expneurol.2016.03.023 27032721

[42] Gowing G , Shelley B, Staggenborg K, et al. Glial cell line-derived neurotrophic factor-secreting human neural progenitors show long-term survival, maturation into astrocytes, and no tumor formation following transplantation into the spinal cord of immunocompromised rats. Neuroreport. 2014;25(6):367-372. 10.1097/WNR.0000000000000092 24284956 PMC3969154

[43] Baloh RH , Johnson JP, Avalos P, et al. Transplantation of human neural progenitor cells secreting GDNF into the spinal cord of patients with ALS: a phase 1/2a trial. Nat Med. 2022;28(9):1813-1822. 10.1038/s41591-022-01956-3 36064599 PMC9499868

[44] Ostenfeld T , Caldwell MA, Prowse KR, et al. Human neural precursor cells express low levels of telomerase in vitro and show diminishing cell proliferation with extensive axonal outgrowth following transplantation. Exp Neurol. 2000;164(1):215-226. 10.1006/exnr.2000.7427 10877932

[45] Di Pierdomenico J , García-Ayuso D, Agudo-Barriuso M, Vidal-Sanz M, Villegas-Pérez MP. Role of microglial cells in photoreceptor degeneration. Neural Regen Res. 2019;14(7):1186-1190. 10.4103/1673-5374.251204 30804243 PMC6425827

[46] Chen WJ , Wu C, Xu Z, et al. Nrf2 protects photoreceptor cells from photo-oxidative stress induced by blue light. Exp Eye Res. 2017;154:151-158. 10.1016/j.exer.2016.12.001 27923559 PMC6054877

[47] Zhang R , Xu M, Wang Y, et al. Nrf2-a promising therapeutic target for defensing against oxidative stress in stroke. Mol Neurobiol. 2017;54(8):6006-6017. 10.1007/s12035-016-0111-0 27696223

[48] Du Y , Li X, Yang D, et al. Multiple molecular pathways are involved in the neuroprotection of GDNF against proteasome inhibitor induced dopamine neuron degeneration in vivo. Exp Biol Med (Maywood). 2008;233(7):881-890. 10.3181/0712-RM-329 18445767

[49] Khan NM , Ahmad I, Haqqi TM. Nrf2/ARE pathway attenuates oxidative and apoptotic response in human osteoarthritis chondrocytes by activating ERK1/2/ELK1-P70S6K-P90RSK signaling axis. Free Radic Biol Med. 2018;116:159-171. 10.1016/j.freeradbiomed.2018.01.013 29339024 PMC5815915

[50] Naguib S , Backstrom JR, Gil M, Calkins DJ, Rex TS. Retinal oxidative stress activates the NRF2/ARE pathway: an early endogenous protective response to ocular hypertension. Redox Biol. 2021;42:101883. 10.1016/j.redox.2021.101883 33579667 PMC8113046

[51] Zhou J , Qu XD, Li ZY, Wei-Ji, Liu Q, Ma Y-H, He J-J. Salvianolic acid B attenuates toxin-induced neuronal damage via Nrf2-dependent glial cells-mediated protective activity in Parkinson’s disease models. PLoS One. 2014;9(7):e101668. 10.1371/journal.pone.0101668 24991814 PMC4081637

[52] Wang Y , Gao L, Chen J, et al. Pharmacological modulation of Nrf2/HO-1 signaling pathway as a therapeutic target of Parkinson’s disease. Front Pharmacol. 2021;12:757161. 10.3389/fphar.2021.757161 34887759 PMC8650509

[53] Wang J , Zhao J, Cui X, et al. The molecular chaperone sigma 1 receptor mediates rescue of retinal cone photoreceptor cells via modulation of NRF2. Free Radic Biol Med. 2019;134:604-616. 10.1016/j.freeradbiomed.2019.02.001 30743048 PMC6619428

[54] Hauck SM , Kinkl N, Deeg CA, et al. GDNF family ligands trigger indirect neuroprotective signaling in retinal glial cells. Mol Cell Biol. 2006;26(7):2746-2757. 10.1128/MCB.26.7.2746-2757.2006 16537917 PMC1430306

[55] Koeberle PD , Bähr M. The upregulation of GLAST-1 is an indirect antiapoptotic mechanism of GDNF and neurturin in the adult CNS. Cell Death Differ. 2008;15(3):471-483. 10.1038/sj.cdd.4402281 18064044

[56] Campochiaro PA , Mir TA. The mechanism of cone cell death in retinitis pigmentosa. Prog Retin Eye Res. 2018;62:24-37. 10.1016/j.preteyeres.2017.08.004 28962928

[57] Murakami Y , Nakabeppu Y, Sonoda KH. Oxidative stress and microglial response in retinitis pigmentosa. Int J Mol Sci. 2020;21(19):7170. 10.3390/ijms21197170 32998461 PMC7583782

[58] Pinilla I , Maneu V, Campello L, et al. Inherited retinal dystrophies: role of oxidative stress and inflammation in their physiopathology and therapeutic implications. Antioxidants (Basel). 2022;11(6):1086. 10.3390/antiox11061086 35739983 PMC9219848

[59] Campello L , Kutsyr O, Noailles A, et al. New Nrf2-inducer compound ITH12674 slows the progression of retinitis pigmentosa in the mouse model rd10. Cell Physiol Biochem. 2020;54(1):142-159. 10.33594/000000210 32028545

[60] Nagar S , Noveral SM, Trudler D, et al. MEF2D haploinsufficiency downregulates the NRF2 pathway and renders photoreceptors susceptible to light-induced oxidative stress. Proc Natl Acad Sci USA. 2017;114(20):E4048-E4056. 10.1073/pnas.1613067114 28461502 PMC5441815

[61] Nakagami Y. Nrf2 is an attractive therapeutic target for retinal diseases. Oxid Med Cell Longev. 2016;2016:7469326. 10.1155/2016/7469326 27818722 PMC5080482

[62] Zhou C , Luo D, Xia W, et al. Nuclear factor (erythroid-derived 2)-like 2 (Nrf2) contributes to the neuroprotective effects of histone deacetylase inhibitors in retinal ischemia-reperfusion injury. Neuroscience. 2019;418:25-36. 10.1016/j.neuroscience.2019.08.027 31442569

[63] Florey O , Kim SE, Sandoval CP, Haynes CM, Overholtzer M. Autophagy machinery mediates macroendocytic processing and entotic cell death by targeting single membranes. Nat Cell Biol. 2011;13(11):1335-1343. 10.1038/ncb2363 22002674 PMC3223412

[64] Chaitin MH , Hall MO. Defective ingestion of rod outer segments by cultured dystrophic rat pigment epithelial cells. Invest Ophthalmol Vis Sci. 1983;24(7):812-820. 6345445

[65] Martinez J , Almendinger J, Oberst A, et al. Microtubule-associated protein 1 light chain 3 alpha (LC3)-associated phagocytosis is required for the efficient clearance of dead cells. Proc Natl Acad Sci USA. 2011;108(42):17396-17401. 10.1073/pnas.1113421108 21969579 PMC3198353

[66] Kim JY , Zhao H, Martinez J, et al. Noncanonical autophagy promotes the visual cycle. Cell. 2013;154(2):365-376. 10.1016/j.cell.2013.06.012 23870125 PMC3744125

[67] Gray M , Botelho RJ. Phagocytosis: hungry, hungry cells. Methods Mol Biol. 2017;1519:1-16. 10.1007/978-1-4939-6581-6_1 27815869

[68] Levin R , Grinstein S, Canton J. The life cycle of phagosomes: formation, maturation, and resolution. Immunol Rev. 2016;273(1):156-179. 10.1111/imr.12439 27558334

[69] Henson PM. Cell removal: efferocytosis. Annu Rev Cell Dev Biol. 2017;33:127-144. 10.1146/annurev-cellbio-111315-125315 28613937

[70] Lancaster CE , Ho CY, Hipolito VEB, Botelho RJ, Terebiznik MR. Phagocytosis: What’s on the menu? Biochem Cell Biol. 2019;97(1):21-29. 10.1139/bcb-2018-0008 29791809

[71] Huynh KK , Eskelinen EL, Scott CC, et al. LAMP proteins are required for fusion of lysosomes with phagosomes. EMBO J. 2007;26(2):313-324. 10.1038/sj.emboj.7601511 17245426 PMC1783450

[72] Cheng XT , Xie YX, Zhou B, et al. Revisiting LAMP1 as a marker for degradative autophagy-lysosomal organelles in the nervous system. Autophagy. 2018;14(8):1472-1474. 10.1080/15548627.2018.1482147 29940787 PMC6103665

[73] Chiang CK , Tworak A, Kevany BM, et al. Quantitative phosphoproteomics reveals involvement of multiple signaling pathways in early phagocytosis by the retinal pigmented epithelium. J Biol Chem. 2017;292(48):19826-19839. 10.1074/jbc.M117.812677 28978645 PMC5712622

[74] Rodríguez-Muela N , Hernández-Pinto AM, Serrano-Puebla A, et al. Lysosomal membrane permeabilization and autophagy blockade contribute to photoreceptor cell death in a mouse model of retinitis pigmentosa. Cell Death Differ. 2015;22(3):476-487. 10.1038/cdd.2014.203 25501597 PMC4326579

[75] Ferguson TA , Green DR. Autophagy and phagocytosis converge for better vision. Autophagy. 2014;10(1):165-167. 10.4161/auto.26735 24220227 PMC4028322

[76] Yao J , Jia L, Feathers K, et al. Autophagy-mediated catabolism of visual transduction proteins prevents retinal degeneration. Autophagy. 2016;12(12):2439-2450. 10.1080/15548627.2016.1238553 27753525 PMC5173283

[77] Chen Y , Sawada O, Kohno H, et al. Autophagy protects the retina from light-induced degeneration. J Biol Chem. 2013;288(11):7506-7518. 10.1074/jbc.M112.439935 23341467 PMC3597791

[78] Zhou Z , Doggett TA, Sene A, Apte RS, Ferguson TA. Autophagy supports survival and phototransduction protein levels in rod photoreceptors. Cell Death Differ. 2015;22(3):488-498. 10.1038/cdd.2014.229 25571975 PMC4326583

[79] Liebl MP , Meister SC, Frey L, et al. Robust LC3B lipidation analysis by precisely adjusting autophagic flux. Sci Rep. 2022;12(1):79. 10.1038/s41598-021-03875-8 34996966 PMC8742033

[80] Das G , Shravage BV, Baehrecke EH. Regulation and function of autophagy during cell survival and cell death. Cold Spring Harb Perspect Biol. 2012;4(6):a008813. 10.1101/cshperspect.a008813 22661635 PMC3367545

[81] Wang S , Girman S, Lu B, et al. Long-term vision rescue by human neural progenitors in a rat model of photoreceptor degeneration. Invest Ophthalmol Vis Sci. 2008;49(7):3201-3206. 10.1167/iovs.08-1831 18579765 PMC3055787

[82] Gardiner KL , Cideciyan AV, Swider M, et al. Long-term structural outcomes of late-stage RPE65 gene therapy. Mol Ther. 2020;28(1):266-278. 10.1016/j.ymthe.2019.08.013 31604676 PMC6951840

[83] Domènech EB , Marfany G. The relevance of oxidative stress in the pathogenesis and therapy of retinal dystrophies. Antioxidants (Basel). 2020;9(4). 10.3390/antiox9040347 PMC7222416 32340220

[84] Jadeja RN , Martin PM. Oxidative stress and inflammation in retinal degeneration. Antioxidants (Basel). 2021;10(5):790. 10.3390/antiox10050790 34067655 PMC8156590

[85] Rodríguez ML , Pérez S, Mena-Mollá S, Desco MC, Ortega AL. Oxidative stress and microvascular alterations in diabetic retinopathy: future therapies. Oxid Med Cell Longev. 2019;2019:4940825. 10.1155/2019/4940825 31814880 PMC6878793

[86] Yumnamcha T , Guerra M, Singh LP, Ibrahim AS. Metabolic dysregulation and neurovascular dysfunction in diabetic retinopathy. Antioxidants (Basel). 2020;9(12):1244. 10.3390/antiox9121244 33302369 PMC7762582

[87] Jones MK , Lu B, Chen DZ, et al. In vitro and in vivo proteomic comparison of human neural progenitor cell-induced photoreceptor survival. Proteomics. 2019;19(3):e1800213. 10.1002/pmic.201800213 30515959 PMC6422354

[88] Tenenbaum L , Humbert-Claude M. Glial cell line-derived neurotrophic factor gene delivery in parkinson’s disease: a delicate balance between neuroprotection, trophic effects, and unwanted compensatory mechanisms. Front Neuroanat. 2017;11:29. 10.3389/fnana.2017.00029 28442998 PMC5385337

[89] Cintrón-Colón AF , Almeida-Alves G, Boynton AM, Spitsbergen JM. GDNF synthesis, signaling, and retrograde transport in motor neurons. Cell Tissue Res. 2020;382(1):47-56. 10.1007/s00441-020-03287-6 32897420 PMC7529617

[90] Kwon W , Freeman SA. Phagocytosis by the retinal pigment epithelium: recognition, resolution, recycling. Front Immunol. 2020;11:604205. 10.3389/fimmu.2020.604205 33281830 PMC7691529

[91] Cuenca N , Fernandez-Sanchez L, McGill TJ, et al. Phagocytosis of photoreceptor outer segments by transplanted human neural stem cells as a neuroprotective mechanism in retinal degeneration. Invest Ophthalmol Vis Sci. 2013;54(10):6745-6756. 10.1167/iovs.13-12860 24045996

[92] Cao J , Murat C, An W, et al. Human umbilical tissue-derived cells rescue retinal pigment epithelium dysfunction in retinal degeneration. Stem Cells. 2016;34(2):367-379. 10.1002/stem.2239 26523756

[93] Nishimura Y , Hara H, Kondo M, Hong S, Matsugi T. Oxidative stress in retinal diseases. Oxid Med Cell Longev. 2017;2017:4076518. 10.1155/2017/4076518 28424744 PMC5382422

[94] Ravera S , Caicci F, Degan P, et al. Inhibitory action of antidiabetic drugs on the free radical production by the rod outer segment ectopic aerobic metabolism. Antioxidants (Basel). 2020;9(11):1133. 10.3390/antiox9111133 33203090 PMC7696108

[95] Pinazo-Durán MD , Zanón-Moreno V, Gallego-Pinazo R, García-Medina JJ. Oxidative stress and mitochondrial failure in the pathogenesis of glaucoma neurodegeneration. Prog Brain Res. 2015;220:127-153. 10.1016/bs.pbr.2015.06.001 26497788

[96] Piano I , Corsi F, Polini B, Gargini C. Nutraceutical molecules slow down retinal degeneration, in Tvrm4 mice a model of retinitis pigmentosa, by genetic modulation of anti-oxidant pathway. Front Neurosci. 2022;16:868750. 10.3389/fnins.2022.868750 35516813 PMC9063314

[97] Zhang X , Girardot PE, Sellers JT, et al. Wheel running exercise protects against retinal degeneration in the I307N rhodopsin mouse model of inducible autosomal dominant retinitis pigmentosa. Mol Vis. 2019;25:462-476. 31523123 PMC6707757

[98] Georgievska B , Kirik D, Björklund A. Aberrant sprouting and downregulation of tyrosine hydroxylase in lesioned nigrostriatal dopamine neurons induced by long-lasting overexpression of glial cell line derived neurotrophic factor in the striatum by lentiviral gene transfer. Exp Neurol. 2002;177(2):461-474. 10.1006/exnr.2002.8006 12429192

[99] Eggers R , Hendriks WT, Tannemaat MR, et al. Neuroregenerative effects of lentiviral vector-mediated GDNF expression in reimplanted ventral roots. Mol Cell Neurosci. 2008;39(1):105-117. 10.1016/j.mcn.2008.05.018 18585464

[100] Touchard E , Heiduschka P, Berdugo M, et al. Non-viral gene therapy for GDNF production in RCS rat: the crucial role of the plasmid dose. Gene Ther. 2012;19(9):886-898. 10.1038/gt.2011.154 21993171

[101] Inana G , Murat C, An W, et al. RPE phagocytic function declines in age-related macular degeneration and is rescued by human umbilical tissue derived cells. J Transl Med. 2018;16(1):63. 10.1186/s12967-018-1434-6 29534722 PMC5851074

