# Supplementary figures and images for: Human Neural Progenitors Expressing GDNF Enhance Retinal Protection in a Rodent Model of Retinal Degeneration
Source: Stem Cells Transl Med. 2023 Oct 3;12(11):727–44. doi: 10.1093/stcltm/szad054 (PMC10630082; doi:10.1093/stcltm/szad054)

## Slide 1
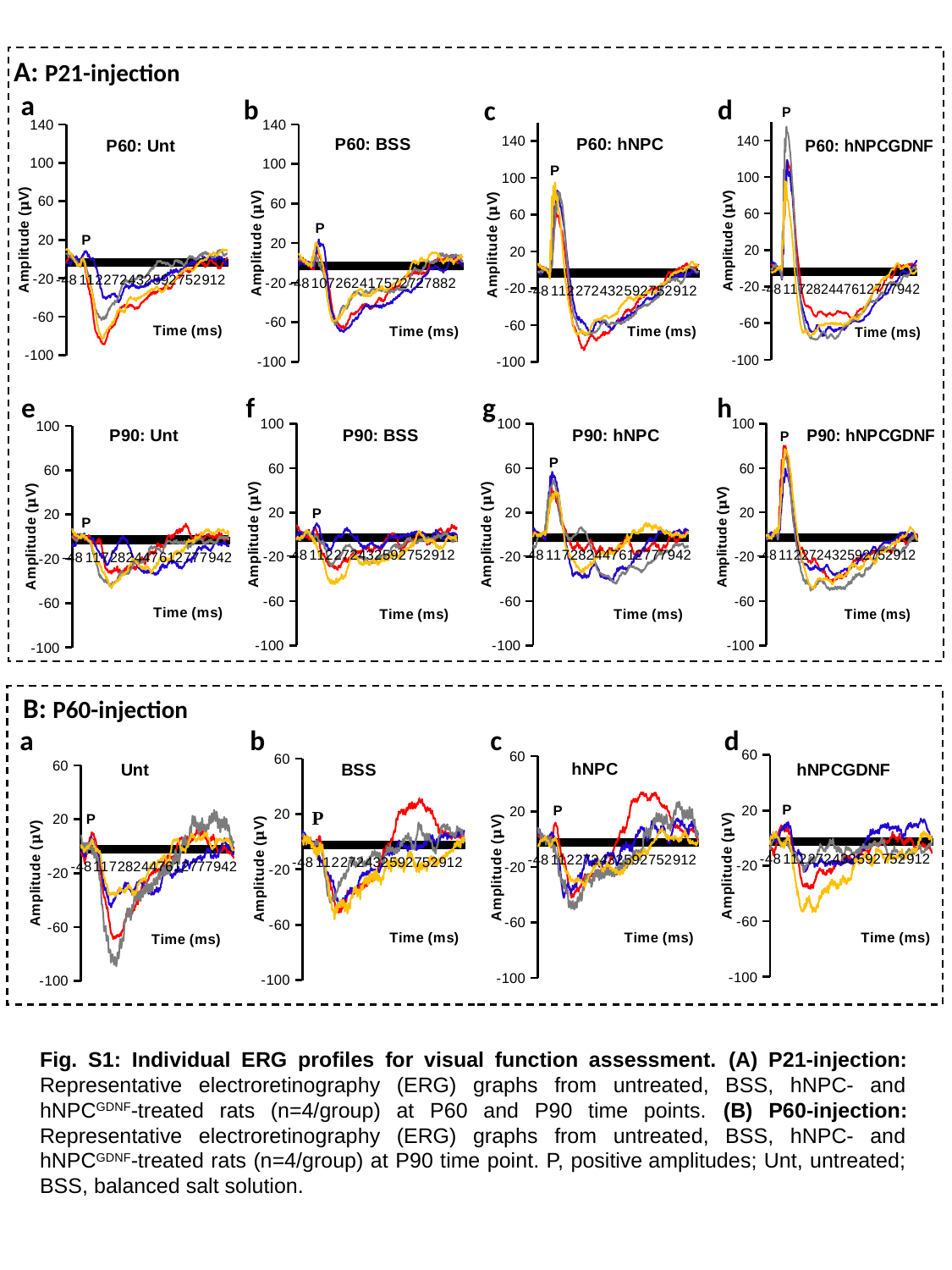

## Slide 2
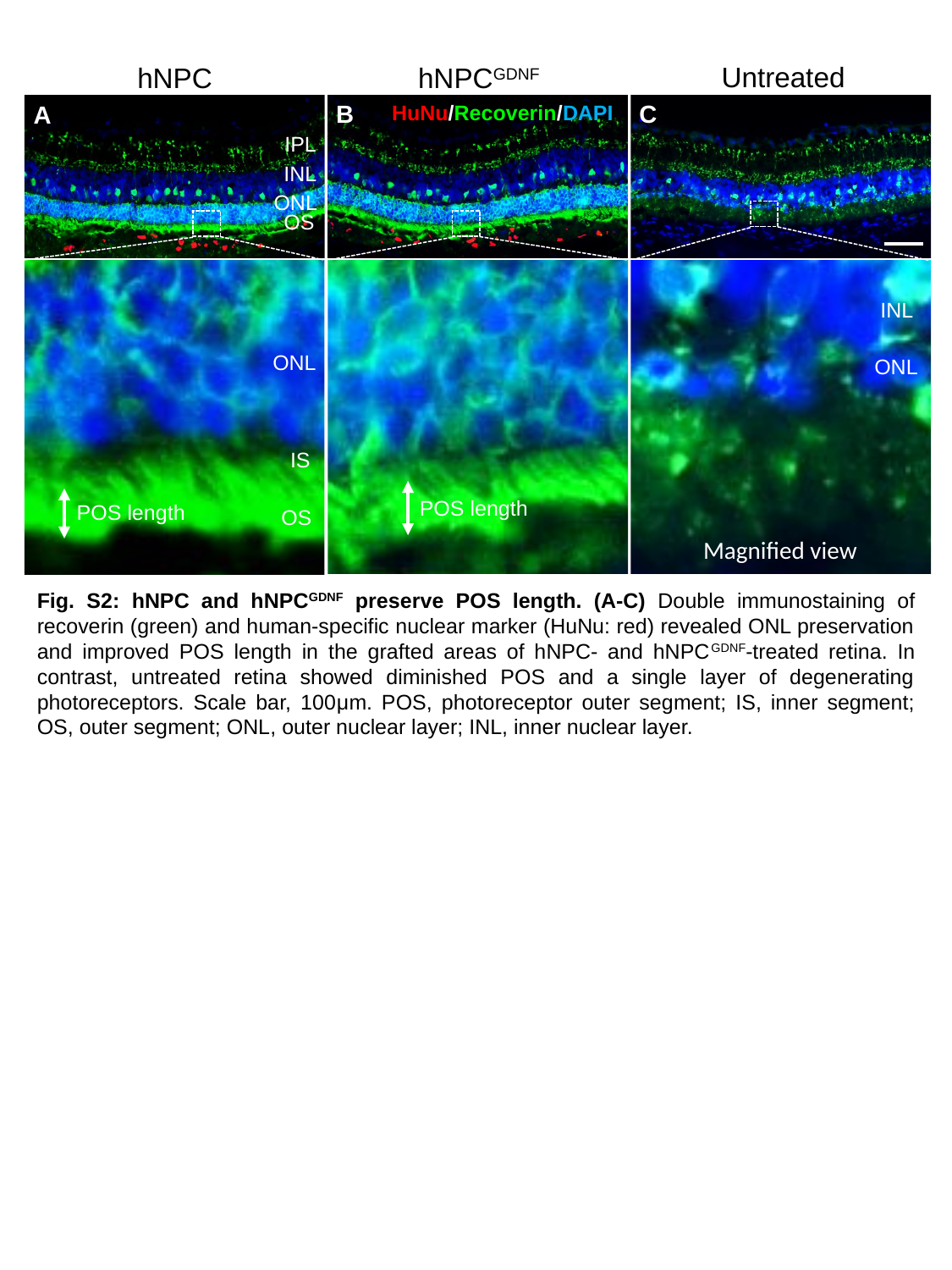

## Slide 3
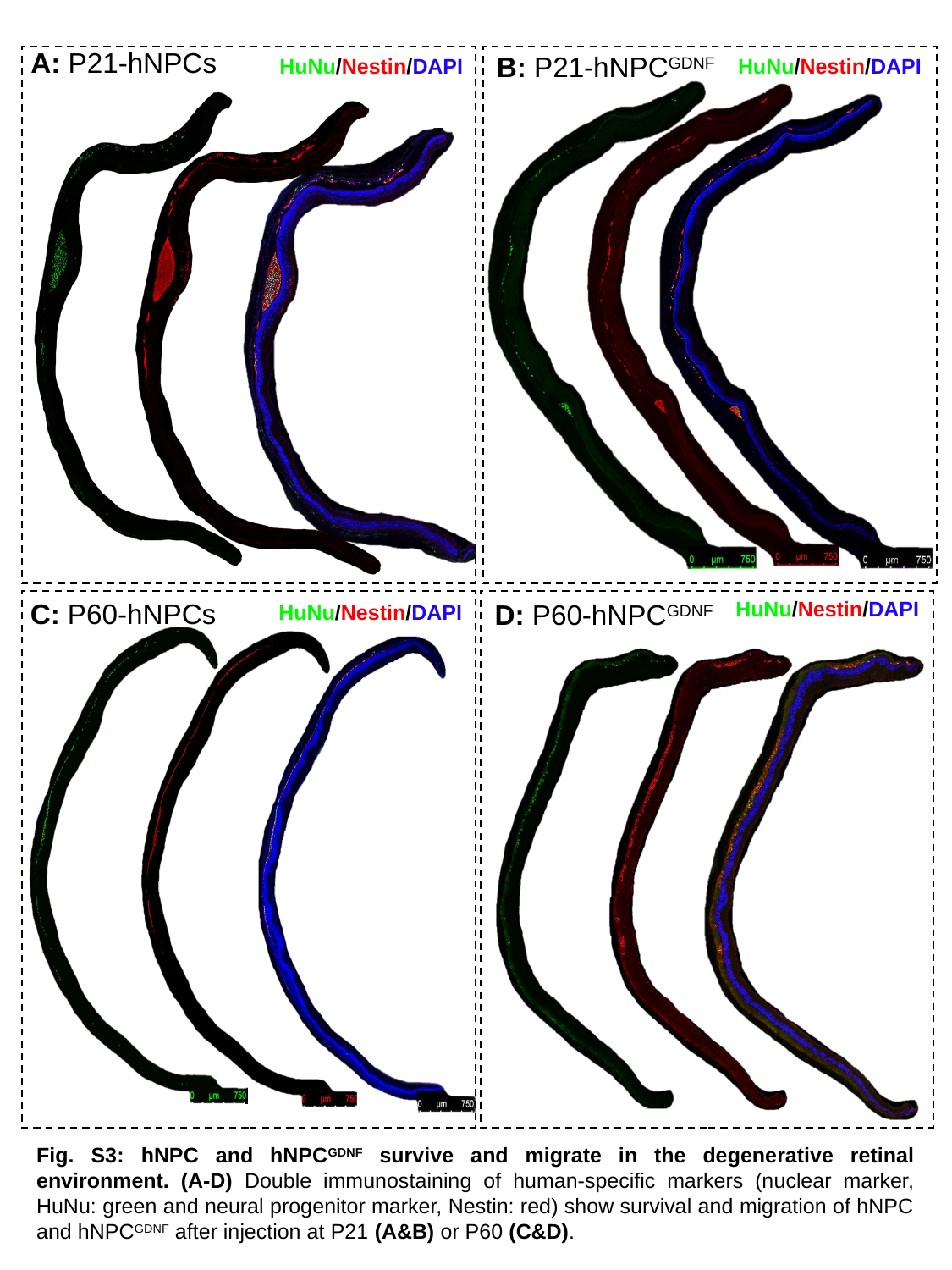

## Slide 4
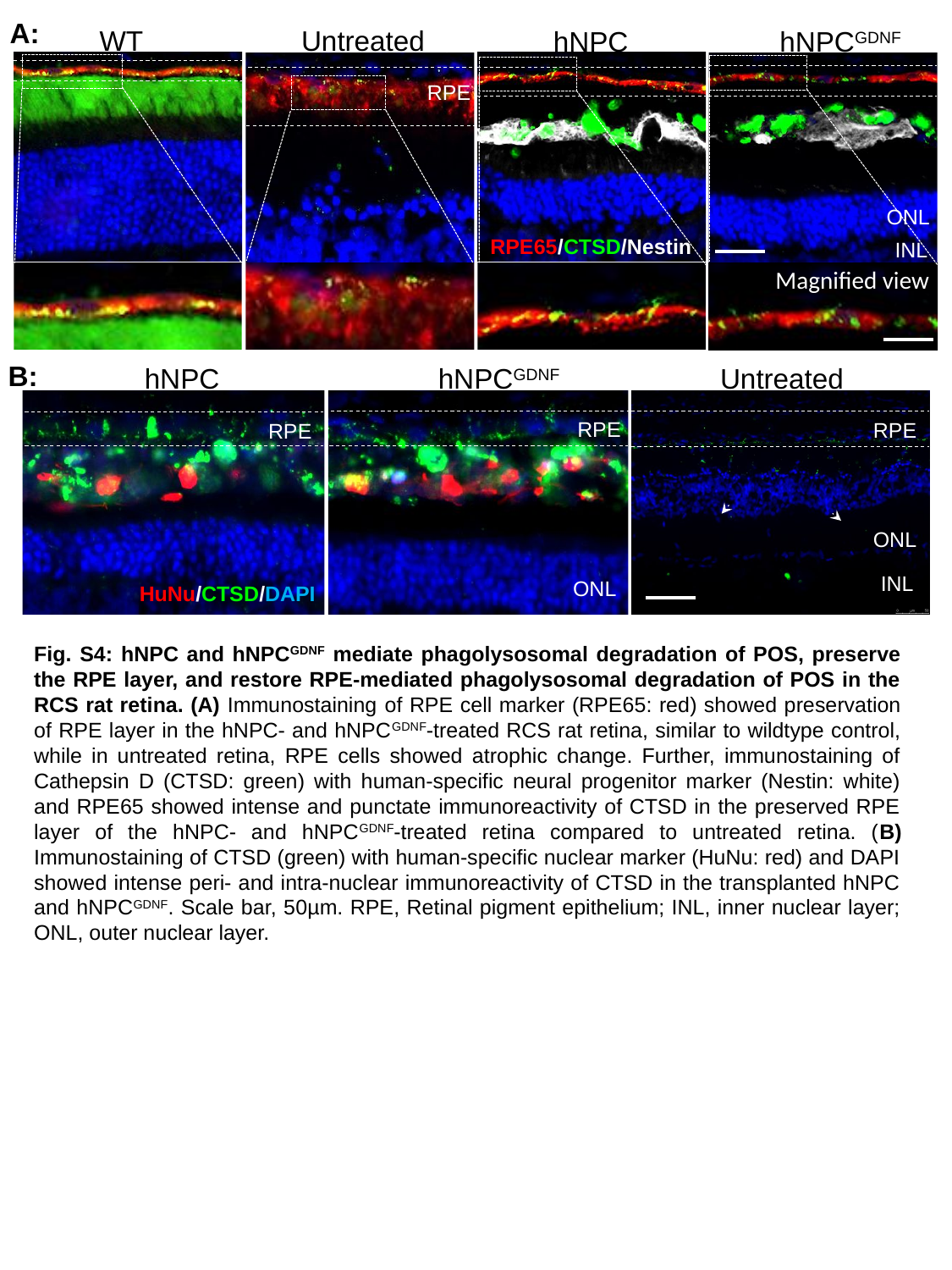

Supplement: szad054_suppl_Supplementary_Figures [file szad054_suppl_supplementary_figures.pptx]
