# Supplementary material for: Human Neural Progenitors Expressing GDNF Enhance Retinal Protection in a Rodent Model of Retinal Degeneration
Source: Stem Cells Transl Med. 2023 Oct 3;12(11):727–44. doi: 10.1093/stcltm/szad054 (PMC10630082; doi:10.1093/stcltm/szad054)
Supplement: szad054_suppl_Supplementary_Table [file szad054_suppl_supplementary_table.docx]

**Supplemental Table 1:** Antibodies used for IF and WB

| **Antibodies** | **Catalogue No.** | **Vendor** | **Dilution** |
| --- | --- | --- | --- |
| Rabbit anti-Iba1 | 019-197441 | FUJIFILM Wako Chemicals | 1:1000 |
| Rabbit anti-Recoverin | AB5585 | Millipore Sigma | 1:2000 |
| Rabbit anti-cone arrestin | AB15282 | Millipore Sigma | 1:1000 |
| Mouse anti-synaptophysin | MAB368 | Millipore Sigma | 1:2000 |
| Mouse anti-Human Nuclear Marker | MAB1281 | Millipore Sigma | 1:300 |
| Rabbit anti-Human Nestin | ABD69 | Millipore Sigma | 1:2000 |
| Rabbit anti-Glial fibrillary acidic protein | Z0334 | Dako-Agilent | 1:1000 |
| Goat anti-GDNF | AF-212 | R&D System | 1:1000 |
| Rabbit anti-Nrf2 | ab137550 | Abcam | 1:200 |
| Rabbit anti-LAMP1 | 21997-1-AP | Proteintech | 1:1000 |
| Goat- anti-Cathepsin D | sc-6486 | Santa Cruz Biotechnology | 1:100 |
| Rabbit anti-LC3B | NB100-2220 | Novus Biologicals | 1:1000 |
| Rabbit anti-GFR⍺1 | PRS1133 | Millipore, Sigma | 1:1000 |
| Rabbit anti-GFR⍺2 | sc-7136 | Santa Cruz Biotechnology | 1:500 |
| Mouse anti-Akt | #2920 | Cell Signaling Technology | 1:1000 |
| Rabbit anti-pAKT | #4060 | Cell Signaling Technology | 1:1000 |
| Rabbit anti-pSrc (Tyr527) | #2105 | Cell Signaling Technology | 1:1000 |
| Rabbit anti-pERK | #4370 | Cell Signaling Technology | 1:1000 |
| Rabbit anti-pGSK-3β | #9336 | Cell Signaling Technology | 1:1000 |
| Mouse anti-GAPDH | G8795 | Millipore Sigma | 1:1000 |
| Rabbit anti-GAPDH | 5174 | Cell Signaling Technology | 1:1000 |
| Mouse anti-RPE65 | MAB5428 | Millipore, Sigma |  |

**Supplemental Table 2:** Primers used for qPCR

| ***Gene*** | **Sense (5'-3')** | **Antisense (5'-3')** | **Accession number** |
| --- | --- | --- | --- |
| *r-Becn1* | GCGTCGGGGCCTAAAGAATG | CTCCTGGCTCTCTCCTGGTT | NM_053739.2 |
| *r-Atg5* | ACCTCGGTTTGGCTTGGTTG | AGTATGGCTCTGCTTCTCGTT | NM_001014250.1 |
| *r-Atg7* | AGCCTGTTCATCCAAAGTTCT | CTGTGGTTGCTCAGACGGT | NM_001012097.1 |
| *r-Gapdh* | ACAGCAACTCCCATTCTTCCA | TCCAGGGTTTCTTACTCCTTGG | NM_017008.4 |
| *r-Hif1-α* | GTTTACTAAAGGACAAGTCACC | TTCTGTTTGTTGAAGGGAG | NM_024359.2 |
| *r-HO-1* | GCTCTATCGTGCTCGCATGA | AATTCCCACTGCCACGGTC | NM_012580.2 |
| *r-Keap1* | GGACGGCAACACTGATTC | TTCGTCTCGATCTGGCTCATA | NM_057152.2 |
| *r-Nrf2* | CACATCCAGACAGACACCAGT | CTACAAATGGGAATGTCTCTGC | NM_001399173.1 |
| *r-NQO1* | CAGCGGCTCCATGTACT | GACCTGGAAGCCACAGAAG | NM_017000 |
| *r-SOD1* | AATGTGTCCATTGAAGATCGTGTGA | GCTTCCAGCATTTCCAGTCTTTGTA | NM_017050 |
| *r-SOD2* | AGGGCCTGTCCCATGATGTC | AGAAACCCGTTTGCCTCTACTGAA | NM_017051 |
| *r-SOD3* | GGGTCTGTCCGTACTTCACCAGAG | CTGACATGGTCCAGGTGACAGAG | NM_012880 |
| *r-Pde8a* | CGGAGGTTTTCAGGAAATGA | GGCCAACGGCTTGAAGAT | NM_198767.1 |
